# Receptor dimerization enables ligand discrimination through tunable response heterogeneity

**DOI:** 10.1371/journal.pcbi.1013781

**Published:** 2025-12-03

**Authors:** Assaf Biran, Yaron E. Antebi

**Affiliations:** Department of Molecular Genetics, Weizmann Institute of Science, Rehovot, Israel; Max Delbruck Centre for Molecular Medicine: Max-Delbruck-Centrum fur Molekulare Medizin in der Helmholtz-Gemeinschaft, GERMANY

## Abstract

Signaling pathways enable cells to coordinate collective behaviors by exchanging specific information. Many pathways utilize multiple ligand variants to activate the same intracellular signaling cascade, raising the question of how cells discriminate between these seemingly redundant signals. It has been shown that individual cells can discriminate between signals based on their induced level of activity, temporal dynamics or combinatorial effect. Here, we demonstrate that ligand discrimination could also emerge at the population level. Using mathematical models of ligand-receptor interactions, we examine how response heterogeneity at the population level can encode ligand identity. We introduce a local scaling metric to quantify how variation in pathway components affects the cellular response. Our results reveal that for pathways with dimeric receptors, and more significantly for heterodimeric receptors, biochemical parameters of the ligands control the resulting heterogeneity in the response of a population of cells. Furthermore, we show that the population-level heterogeneity encodes the enzymatic activity of the resulting receptor complex. This suggests a functional advantage for utilizing heterodimeric receptor complexes in pathways acting across a population of cells, such as the type I interferon pathway, which shows several of the characteristics of our model. This contrasts to juxtacrine pathways, such as Notch, that do not act at the population level and use a single component receptor. Overall, our findings highlight a novel mechanism by which receptor architecture enables cells to encode ligand-specific information through population-level heterogeneity, offering insights into immune regulation, tissue development, and synthetic biology.

## Author summary

Cells constantly exchange messages to coordinate their behavior, during both development and in maintaining health. Many of these messages, proteins called ligands, use the same cellular machinery but nevertheless lead to very different outcomes. Most research has focused on how single cells can distinguish these messages. In this study, we asked a different question: can ligand identity be encoded in how varied the responses are across an entire population of cells? Controlling population variability can strongly influence the collective behavior of a tissue. Using mathematical models of receptor–ligand interactions, we found that the architecture of the receptor complex itself plays a key role. Simple one-part receptors respond proportionally to the different ligands, so population variability cannot be tuned by the signal. In contrast, receptors built from two parts, especially when the parts differ as in pathways like type I interferon or TGF-β/BMP, allow ligand identity and relative amount to shape the spread of responses across cells without changing the average level. This population-level “fingerprint” could influence immune defense, tissue development, or drug responses, and suggests new ways to design therapies or synthetic systems that harness variability rather than simply suppressing it.

## Introduction

**Fig 1:**
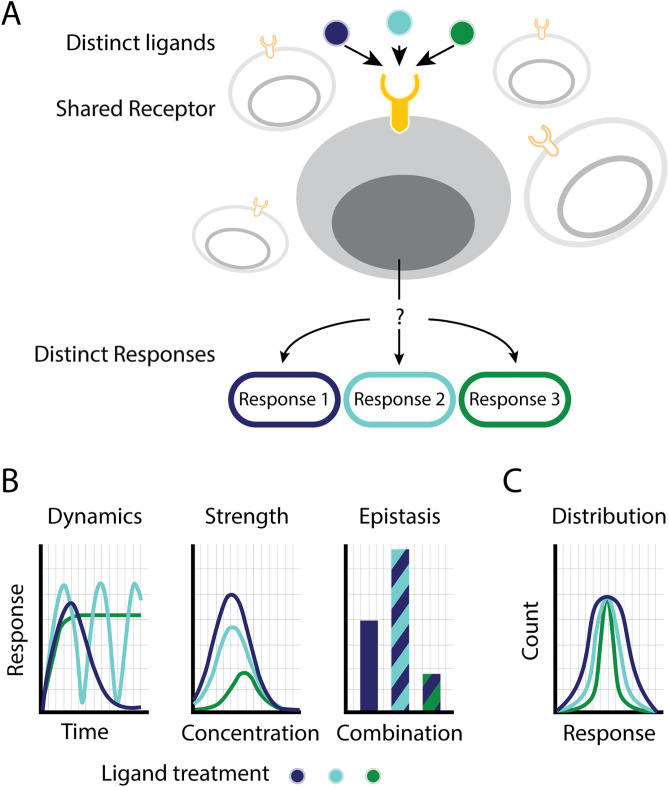
Ligand discrimination in multi-ligand pathways. (A) Signaling pathways comprise multiple ligands that bind the same receptors on the membrane of cells. This provokes the question of whether and how cells in a population discriminate between the different ligands of the same pathway. (B) Several mechanisms have been suggested for ligand discrimination on the single-cell level. In dynamic ligand discrimination, ligands elicit different response dynamics over time (left). Discrimination could also be achieved when ligands activate the pathway with distinct response strength (center). Finally, the epistatic relationship of one ligand with other ligands of the same pathway could result in a ligand-specific response (right). (C) Alternatively, ligand discrimination could occur at the level of the population. In this case, the ligand identity results in a variation of population-level properties, such as heterogeneity across the population of cells. Inducing distinct levels of variability across the tissue, ligands can result in distinct phenotypes.

Intercellular signaling pathways regulate many aspects of multicellular organisms, from development, through homeostasis, to immune responses [1–3]. These processes inherently rely on multiple cells acting together in a coordinated manner [2,3]. To achieve such coordinated behaviors robustly, cells secrete and respond to a wide set of ligands. Interestingly, many signaling pathways, such as type I interferon (IFN), bone morphogenic protein (BMP), transforming growth factor β (TGFβ), fibroblast growth factors (FGF), Notch, and others, show a peculiar feature. Multiple distinct ligands promiscuously interact with shared receptors to activate the same downstream pathway [1,4,5]. In many cases, these distinct ligands give rise to distinct biological outcomes, despite using the same intracellular mediators [6–8] (Fig 1A). Thus, a central challenge in understanding the regulatory capacity of cell-to-cell communication is determining the extent and mechanisms by which cells encode ligand identity and discriminate between seemingly equivalent ligands.

Previous work has demonstrated several different mechanisms for ligand discrimination. In the Notch signaling pathway, different ligands show distinct temporal dynamics that lead to the activation of distinct downstream target genes [8] (Fig 1B, left). A similar mechanism is exhibited by the NF-KB transcription factor, which discriminates between different stimuli based on the different activation dynamics [9]. Alternatively, ligands can be distinguished through their maximal activation level (Fig 1B, middle) [6,10,11]. In the interferon pathway, ligand-specific activity strength can lead to specific expression levels of target genes [12,13] or to activation of different set of genes by activating regulatory elements with distinct affinities [14]. Lastly, it was recently shown that seemingly equivalent ligands could exhibit distinct combinatorial effects [5,15]. Such combinatorial specificity allows different ligand combinations to differentially activate specific target cells [16] (Fig 1B, right). Overall, several signal discrimination mechanisms have been identified that enable individual cells to decode the identity of the signal or signal combinations.

Many processes during development, homeostasis, and regeneration inherently control the population behavior of cells. In these cases, ligand discrimination might not arise at the single-cell level, but rather, ligands could be distinguished by the way they affect the overall population behavior. In particular, a defining trait of biological systems is the inherent stochasticity among cells. Within the same population, cells often express RNA and proteins at different levels [17–19]. This variability has been shown to have important and beneficial functional roles [17]. For example, stochasticity could result in a robust response at the population level [20,21] or allow an efficient way to regulate gene expression and differentiation [18,22]. In single-cell organisms, such as bacteria and yeast, as well as cancer cells, stochasticity in gene expression allows for bet-hedging, where a small, random subpopulation of cells express certain genes that allow them to survive extreme conditions or drug treatments [23–26]. External regulation of the stochasticity within the population has been shown to allow control over the behavior of bacteria and yeast [24,27]. Thus, ligand-dependent control of heterogeneity can have an important biological role in determining cellular responses.

A prominent example of this importance in multicellular organisms is the type I IFN antiviral pathway [3]. The type I IFN pathway is a significant component of the innate immune response. It helps protect against infections and has an immunomodulatory role in cancers [3,12]. Importantly, excess activation of the type I IFN pathway was found to be involved in the initiation and sustainment of autoimmune diseases [28,29]. As such, it is critical for the number of responding cells to be tightly controlled [28]. It was further found that cell-cell variability in this pathway originates from heterogeneity in the amount of receptor expressed [12–14]. This suggests a new type of ligand-specific effect in which cells can be activated at the same average level but with distinct levels of heterogeneity across the population in a ligand-dependent manner (Fig 1C). In this way, different ligands could control the variability of the response within the population, leading to distinct systematic behaviors.

Here we use mathematical models, motivated by the type I IFN pathway, to analyze the capacity of signaling pathways to exhibit ligand-dependent heterogeneity. We formulate and analytically solve models describing several receptor architectures. For each architecture, we analyze the response heterogeneity and determine to what extent ligand identity can attenuate this heterogeneity. We start with models of single subunit receptors and find a robust heterogeneity that cannot be controlled by extracellular ligands. Continuing to study a more complex model of receptors acting through ligand-induced dimerization, we find that heterogeneity depends on the total amount of full complexes that are formed. In this case, ligand parameters can affect and tune the relative heterogeneity. Moreover, we distinguish between homo- and heterodimerizing receptors. Models with homodimerizing receptors show limited sensitivity to ligand characteristics, giving rise to a limited ligand discrimination capacity. Models with heterodimerizing receptors, however, allow for greater control over the response heterogeneity in a ligand-dependent manner. As such, they provide a mechanism for a flexible ligand discrimination capacity. Taken together, our results reveal the impact of ligand identity on the distribution of cell response to stimuli for different receptor architectures. In pathways with multi-component receptor complexes, such as type I IFN and other immunological and developmental pathways, this suggests that specific ligand variants can result in distinct overall heterogeneity in the response that can lead to ligand-specific functional phenotype.

## Results

### Signaling pathways with monomeric receptors give rise to response heterogeneity that is independent of ligand identity

**Fig 2:**
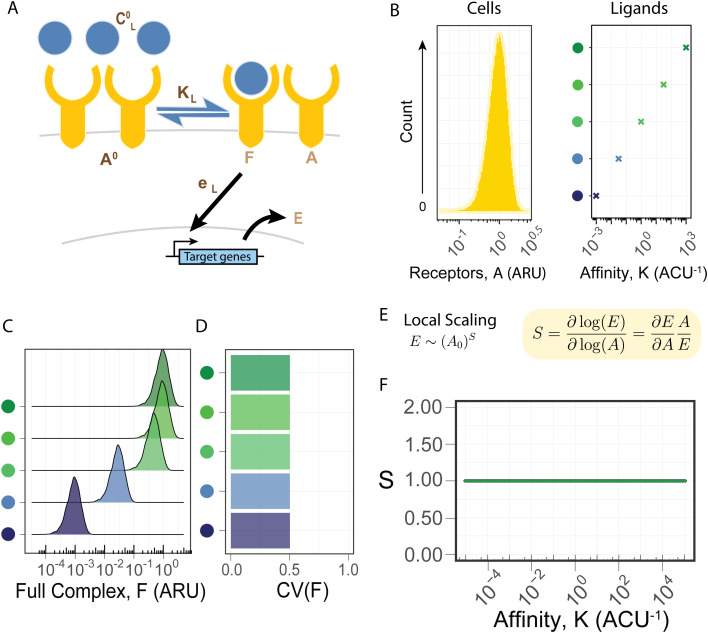
Single-unit receptor architecture does not allow for ligand-dependent heterogeneity. (A) A minimal model for a pathway with a single-unit receptor. Receptors (A) and ligands (C) bind to form full complexes (F) and activate target genes (E). The four model parameters (total receptors, A^0^; ligand concentration, C^0^; affinity, K; and enzymatic efficiency, e) are shown in dark brown, and the three variables are shown in light brown. Parameters that depend on the specific ligand identity are denoted with a subscript L. (B) We consider the response of a population of 10,000 cells with a Gamma distribution of receptors with a mean of one (ARU = Arbitrary Receptor Units) and standard deviation of 0.5 (left) to five ligands with a concentration of one (ACU = Arbitrary Concentration Units) and different affinity values (right). (C, D) The response distribution (C) and the coefficient of variation (D) are plotted for each ligand. (E) The local scaling, defined as the logarithmic derivative, reflects the relative change in the response to relative changes in receptors. While the population level coefficient of variation (CV) depends on the specific receptor level distribution, the scaling is defined at an individual cell level, and is a local measure of the heterogeneity. As the scaling value increases, the population level CV increases as well. (F) The scaling with the single-unit receptor model is constant and independent of the ligand parameter.

We start by considering the basic architecture of a signaling pathway, which consists of a single-unit receptor that binds an extracellular ligand. The formation of a ligand-receptor complex then leads to the induction of a downstream intracellular response. Such architectures are utilized in several biological pathways, such as the Notch signaling pathway [8], and certain G-protein coupled receptor systems, such as the CCR1 chemokine receptor and its ligands [30] (Table 1). Such systems can be described using a minimal mathematical model, where a ligand, denoted by *L*, binds to a receptor, *A,* to form a fully active signaling complex, *F* (Fig 2A). Once a complex is formed, it induces intracellular changes leading to the transcription of target genes. The behavior of this model is governed by four parameters. The total amount of receptors on the cell surface, A^0^, is inherent to the cells and independent of the extracellular environment. In contrast, the concentration of the ligand in the environment, *C*^*0*^, the affinity of the ligand to the receptor, *K*_*L*_, and the rate by which the complex activates the downstream pathway, *e*_*L*_, are properties of the specific ligand used and can be changed accordingly.

**Table 1:** Receptor-Ligand interactions occur with different binding architectures throught biology.

Model	Architecture	Scaling Range	Ligand-Dependent Heterogeneity	Example Pathways
AL	Monomeric receptor	Constant (S = 1)	No	Notch [8]
ALA	Homodimeric receptors with one ligand	Limited (1 < S < 2)	Yes (modest)	TNF [31], NGF [32]
LAAL	Homodimeric receptors with two ligands	Limited (1 < S < 2)	Yes (modest)	FGF [32,33], EGF [34]
ALB	Heterodimeric receptors	Broad (0 < S < 1)	Yes (strong)	IFN [4,35–37], TGFβ [38], BMP [5], Interleukins [39]

To model these pathways, we assume a fast binding-unbinding dynamics on a short timescale, such that the steady state describes the initial immediate response of the pathway, before feedback mechanisms acting on receptor or ligand levels take effect. At steady-state, the model can be specified by the set of ligand-receptor binding-unbinding equations, together with an additional equation for mass conservation of the total receptors (S1 Text). Furthermore, we consider the case of pathways where ligands are soluble and diffusive across the entire volume around the cells, while receptors are bound to the membrane. Thus, the number of ligand molecules significantly surpasses the number of receptors, and binding of ligands to the receptor does not affect their overall concentration in the environment (see supplementary text for more details). In addition, we assume that receptor production and degradation are balanced at steady state. In many signaling pathways, however, receptors undergo ligand-dependent degradation [40–43], with active complexes being removed more rapidly than inactive forms. To address this more general case, we extended our model to include such ligand-dependent receptor turnover and found that the key results remain unchanged (see S1 Text). Solving the equations at steady-state gives rise to the Michaelis-Menten dependence of response on ligand concentration (S1A Fig) [6,10].

Using the analytical solution, we can determine the response heterogeneity and its dependence on ligand identity through its chemical properties. In our analysis we quantify heterogeneity by the coefficient of variation (CV) to normalize the general dependence of standard deviation on rescaling of the distribution. To analyze the heterogeneity, we assume that the media is well mixed, and thus all cells receive the same ligand concentration. We focus on response variability derived from noise in receptor expression levels. This has been shown to account for most of the variability in the IFN type II pathway [44], as well as differential responses and cell fate in the IFN type I pathway [14,45]. We considered a population of 100,000 cells and simulated variable receptor expression levels distributed with a gamma distribution (Fig 2B, left). We examine five different ligands at the same concentration but with different affinities (Fig 2B, right). While the population mean amount of formed complexes is dependent on the ligand parameters (Fig 2C), the heterogeneity is robust across all ligands, regardless of their affinity to the receptor (Fig 2D). These examples suggest that in this model, the response heterogeneity is independent of any biochemical parameter and cannot be adjusted.

We next aimed to determine the robustness of response heterogeneity more systematically and for general distributions of receptors. Using simulations to compute the CV for distinct distributions of receptors with distinct levels of variability is computationally prohibitive [21]. Instead, we pursued a more basic metric, that will hold at the single cell level, for analyzing heterogeneity that is independent of the specific shape of the overall distribution. We consider the local scaling, S, between the response and the amount of receptors in a specific cell (Figs 2E, S1B, S1 Text). This scaling is defined for individual cells, as the relative change in the response (E) for a small relative change in the receptor amount (A_0_) and represents the power law dependence of the response on the amount of receptors (E ~ A_0_^S^). A system with a linear dependence, S=1, will have a similar CV in the response as in the receptors. For a sublinear dependence, S < 1, the CV of the response is reduced compared to the CV of the receptors, while a superlinear dependence, S > 1, results in increased response heterogeneity. We are interested in determining whether S depends on ligand parameters, in which case the response variability will depend on the identity of the ligand. Thus, this local scaling metric allows us to analytically determine the capacity of ligand identity to determine population heterogeneity in the response.

Computing the local scaling metric for the basic model of a single unit receptor, we find that the scaling is identically equal to one, independently of any biochemical parameter (Fig 2F). In particular, the scaling of the response to changes in the receptors is independent of the activity rate, e_L_. This reflects the linear dependence of the response on the receptors (S1 Text) as each receptor binds independently to the ligand, and its contribution to the total complex amount is independent and additive. In the case of monomeric receptors, the scaling does not depend on the parameters and the response heterogeneity reflects only the heterogeneity of the receptors, in agreement with our simulation-based result (Fig 2C, 2D). We then tested the effect of incorporating non-linear dependence between the number of complexes and the response. This could arise, for example, from cooperativity between downstream mediators, which can give rise to power-law dependence. Analyzing this extended model (see Supplementary Information), we find that such non-linearities shift the scaling value away from 1. Importantly, however, the scaling remains independent of ligand biochemical parameters. Thus, while heterogeneity might differ in pathways with non-linear responses, it is still independent of ligand identity and cannot be tuned. We thus find that in a single-unit receptor system, the population distribution does not provide a way to discriminate between ligands.

### In pathways with dimeric receptors, ligand properties can affect the response heterogeneity

**Fig 3:**
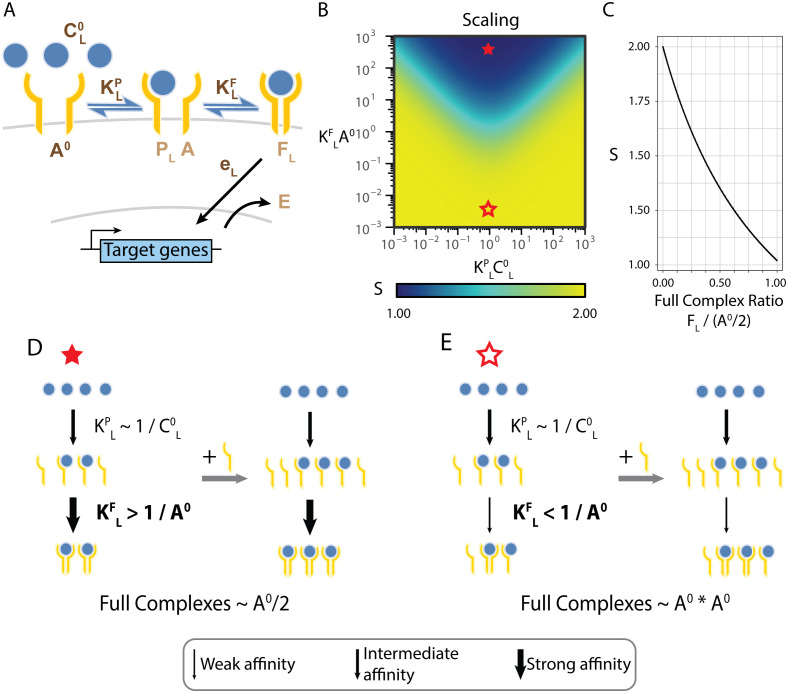
Homodimeric receptor architecture allows for ligand-dependent scaling. (A) A minimal model for a pathway with a homodimeric receptor. A single ligand molecule binds sequentially two receptor subunits to form partial (P) and full (F) complexes and activate target genes. The five model parameters are shown in dark brown, and the four variables are shown in light brown. Parameters that depend on the specific ligand identity are denoted with a subscript L. (B) The model’s scaling (*S*) to changes in the receptor subunit *A*^*0*^ is plotted as model parameters vary. The full and hollow red stars indicate the parameter regimes discussed in D and E, respectively. (C) The dependence between the scaling and the fraction of bound receptors (2*F*_*L*_/*A*^*0*^) is plotted. (D, E) The effect of receptor addition on the response depends on the model’s parameters. Arrow thickness indicates the relative strength of the parameters. (D) When *K*^*P*^_*L*_*C*^*0*^_*L*_*≈1* and *K*^*F*^_*L*_*A*^*0*^*>>1* (full star in B), all free receptor subunits *A* bind to any free partial ligand-receptor complex *P*_*L*_ so that the amount of *P*_*L*_ *is* the limiting factor in complex formation, and *F*_*L*_ is linearly dependent on *A*^*0*^. (E) When *K*^*P*^_*L*_*C*^*0*^_*L*_*≈1,* as before, but *K*^*F*^_*L*_*A*^*0*^*<<1* (hollow star in B), most *P*_*L*_ will be free of receptors. In this case, *F*_*L*_ will be proportional to the product of *A* and *P*_*L*_, and as *P*_*L*_’s amount is proportional to that of *A*, *F*_*L*_ scales quadratically with *A*^*0*^.

Pathways with a single unit receptor are not generally common in mammalian systems. Rather, in most mammalian signaling pathways, receptors are formed from a bound complex with multiple receptor subunits. In some cases, e.g., the NGF pathway and other receptor tyrosine kinases (Table 1), a ligand binds to two equivalent receptor subunits, which form a full signaling complex and initiate the intracellular response [32]. To study this more complex architecture of homodimeric receptors, we extended our model to describe a two-step formation of the signaling complex (S1 Text). Briefly, a ligand denoted by *L* first binds to one receptor subunit, *A,* to form a partial complex *P*_*L*_ with an affinity of *K*^*P*^_*L*_. Once *P*_*L*_ is formed, it can bind a second identical receptor subunit, *A,* to form the full, trimeric complex *F*_*L*_, with an affinity *K*^*F*^_*L*_. The full complex, *F*_*L*_, can then induce the expression of target genes, *E*, at a rate *e*_*L*_ (Fig 3A). In this model, the ligand-dependent parameters are the concentration of the ligand, *C*_*L*_, its affinity to the receptor subunit, *K*^*P*^_*L*_, the affinity of the partial complex to the receptor subunit, *K*^*F*^_*L*_, and the activation rate of the full complex, *e*_*L*_. Together, these four parameters define a specific signaling environment for the cells. The configuration of the cells is defined by the level of total receptor subunits, *A*^*0*^, which does not depend on the ligand identity. As before, we assume that ligands concentration is not reduced upon binding of the receptors. This extended version of the model allows us to analyze the more complex case of dimeric receptors.

We next used the model to determine the scaling of the response to changes in receptor levels. We start by focusing on binding-unbinding parameters, keeping the rate of downstream activation fixed, *e*_*L*_ = 1, for all ligands. Using dimensional analysis (S1 Text), we find that the behavior of the system depends only on the dimensionless quantities *K*^*P*^_*L*_*C*_*L*_ and K^F^_L_*A*. This is consistent with previous studies of this model, which have shown that the ligands’ concentration can be compensated for by its affinity to the free subunit, *K*^*P*^_*L*_, and that the amount of receptors can be compensated for by the partial complex’ affinity to the free subunit, *K*^*F*^_*L*_ [46,47]. Solving the response in steady-state gives rise to a non-monotonic dependence on ligand concentration (S2A Fig). Using the expression for the response, we analytically solved for the local scaling (Fig 3B, 3C, S1 Text). In contrast to the single-unit receptor model, we find that the scaling depends on ligand parameters but has a limited range, showing a fold-change of two.

The model can further determine the molecular mechanism allowing controlled heterogeneity in this context and gaining an intuition for its limited range. To this end, we looked more closely at the different parameter regimes of the model. We focus on two ligand parameter regimes exhibiting distinct scalings, with one ligand having the lowest possible scaling (S=1, linear dependence) and the other the highest (S=2, quadratic dependence) (Fig 3B, filled and hollow star). The first regime (marked by a filled star) is characterized by high trimeric affinity (*K*^*F*^_*L*_*A*^*0*^≫* 1)* and a concentration of ligands around the EC50 for the formation of partial complexes *K*^*P*^_*L*_*C*_*L*_* ≈ 1*. In this regime, the equation for the dependence of the activity on the receptors simplifies and becomes linear (S1 Text). Intuitively, if we start with free receptors, the first binding reaction is at its EC50 and results in half of the receptor forming partial complexes while the other half of them will remain free. Since the formation of the full trimeric complex is saturated (*K*^*F*^_*L*_*A*^*0*^≫*  1*), all partial complexes will then bind with the available receptors. Overall, most receptor subunits will be paired up efficiently to form full complexes, resulting in a linear dependence of the total amount of complexes, *F*_*L*_, on the amount of receptor subunits, *A*^*0*^ (Fig 3D). In this parameter regime the dimeric receptor model is analogous to the monomeric receptor model.

In the second regime (hollow star), on the other hand, the full complex affinity is low (*K*^*F*^_*L*_*A*^*0*^*<<1*). In this case, the second step reaction, forming the full complex, has low affinity and is far from saturation, resulting in many available free partial complexes and free receptors. In this regime, forming a few full complexes does not significantly change the amount of receptors and partial complexes. In other words, this regime can be analyzed as a binding reaction with no depletion of the components. In such conditions, the amount of full complexes will be proportional to the product of the amount of receptors with the amount of partial complexes. Considering the EC50 concentration for partial complex formation, the amount of partial complexes is proportional to the amount of receptors. Thus, the overall amount of full complexes, *F*, scales quadratically with the amount of receptors, A^0^ (Fig 3E). This can also be shown analytically to hold, even for a more general range of parameters (see S1 Text). We see that the dependence of the amount of full complexes on the amount of receptors shifts from linear in the first regime to quadratic in the second. Accordingly, the local scaling of the full complex, with respect to receptor levels, varies from one to two, reflecting a corresponding change in the heterogeneity.

Some pathways have a similar architecture of dimeric receptors, but with different ligand-receptor stoichiometry (Table 1). For example, the signaling complex for the FGF pathway is composed of two receptor subunits and two ligands. We have analyzed this variant (the LAAL model, see S1 Text) and found that the main results remain similar. The scaling depends on ligand-related biochemical parameters but varies within a limited range between S=1 and S=2 (S2B-S2D Fig). We thus conclude that dimeric receptors enable tunability of the response heterogeneity across biologically relevant pathway architectures.

### Response heterogeneity encodes the relative effective activity parameter of the ligand

We next studied what ligand properties are important for controlling the heterogeneity. We found that while the scaling has a complex dependence on the parameters, it can be written as a simple function of the full trimeric complex, F_L_, with no additional dependence on parameters (Fig 3C, S1 Text). When more complexes are formed, the system becomes less sensitive to changes in receptors, and the scaling is reduced. We emphasize that this inverse dependence emerges from the architecture of the pathway, as it does not occur for the simpler, monomeric receptor architecture.

The direct dependence of the scaling on F_L_ has an important implication. While the overall response depends both on the amount of complexes as well as the activity rate, *e*, the scaling does not depend on the activity rate (S1 Text). Hence, if cells are exposed to ligands with distinct activity parameters, then for the same overall population mean response, the population will necessarily exhibit distinct complexes amounts which would mean a difference in the response variability. In this way, a dimeric system encodes the activity rate of ligands into variability in the response, and thus the population response can differ between ligands, even when the population mean response is the same.

**Fig 4:**
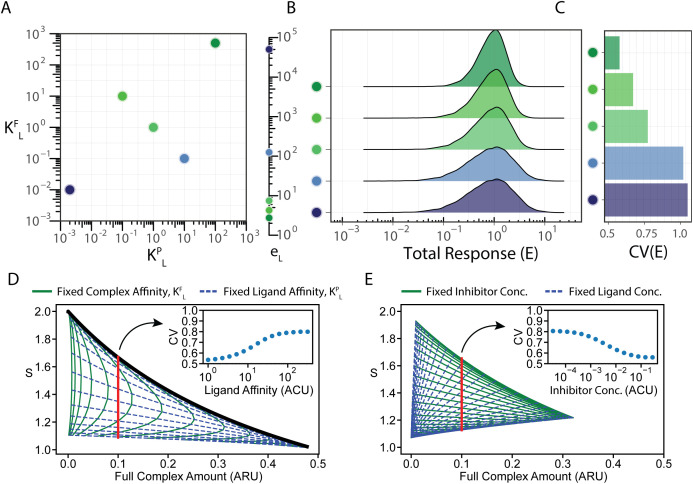
Ligand efficiency is encoded in the heterogeneity of the response. (A) We consider five ligands with different affinities (*K*^*F*^_*L*_, *K*^*P*^_*L*_) and activation rates (*e*_*L*_). (B, C) We simulated the response of a population of 100,000 cells to the five ligands. Cells were assumed to express receptors with a Gamma distribution of unit mean and 0.5 standard deviations. To directly compare the variability, the parameters were chosen such that the population mean response equals 1. The response distribution (B) and the coefficient of (C) are plotted for each ligand. (D) The scaling was simulated for ligands with different chemical parameters in the presence of a zero-activity inhibitor. A green full line represents some fixed complex affinity, K^F^_L_, with varying ligand affinity, K^P^_L_. A blue dashed line represents some fixed ligand affinity with varying complex affinity. The black line represents the dependence without an inhibitor (cf Fig 3C). A set of affinity parameters resulting in a fixed response level (red line) was selected, and the CV of the response was computed for a population of cells as before (inset). (E) The scaling was simulated at varying ligand and inhibitor concentrations. A green full line represents some fixed inhibitor concentration with varying ligand concentrations. A blue dashed line represents some fixed ligand concentrations with fixed inhibitor concentrations. A set of relative concentrations resulting in a fixed response level (red line) was selected, and the CV of the response was computed for a population of cells as before (inset). ARU = Arbitrary Receptor Units, ACU = Arbitrary Concentration Units.

This population-level effect can be demonstrated by using the model to simulate the response of a population of cells under ligands with distinct parameters. To this end, we simulated a population of 100,000 cells with gamma-distributed expression of receptor *A*, as before. We analyzed the response’s distribution under the induction of five different ligands with different parameters (Fig 4A). For each ligand, we chose the parameters such that the population mean response is identical while the activity rate differs. Calculating the CV, as before, we find that the different ligands induce a different level of heterogeneity in the response, even though they have the same population mean response (Fig 4B, 4C). As we observed for the scaling metric, the ratio between the CV across different ligands is limited to twofold. Furthermore, we see that the standard deviation of the response can be closely approximated by the product of the receptor standard deviation by response scaling (S2E Fig). Thus, ligand-dependent variability can be seen for simulated distributions of cells.

An extreme example of ligands with different activity rates is the case of competitive inhibitors. These inhibitors can bind the receptor but do not activate the downstream pathway. In our model, this is the case where e_L_ = 0. Since for this case, the pathway remains completely off, we decided to analyze the case of a combination of different ligands with an inhibitor. As this two-ligand model does not lend itself to an analytic solution, we simulated the system using the EQTK toolbox [48,49]. We varied the two affinity parameters for the activating ligand while keeping the inhibitor fixed. We find that the presence of an inhibitor can break the direct dependence between S and F_L_ (Fig 4D). Distinct ligands show distinct scaling properties, even with the same efficiency. When simulating the response of a population of cells, we can see that, indeed the CV depends on the ligand affinity (Fig 4D, inset). Finally, we note that changing the concentration of the inhibitor and the signal also results in different scalings for the same number of complexes (Fig 4E). In this way, competitive inhibitors provide a mechanism to tune the heterogeneity of a response. If we vary the amount of inhibitor and signal such that the number of complexes stays constant, we see that the heterogeneity across the population is reduced as the amount of inhibitor increases (Fig 4E, inset). Our results demonstrate a new role for competitive inhibitors in shaping the heterogeneity of the response to signals.

### Heteromeric receptor systems allow general tuning of response heterogeneity

**Fig 5:**
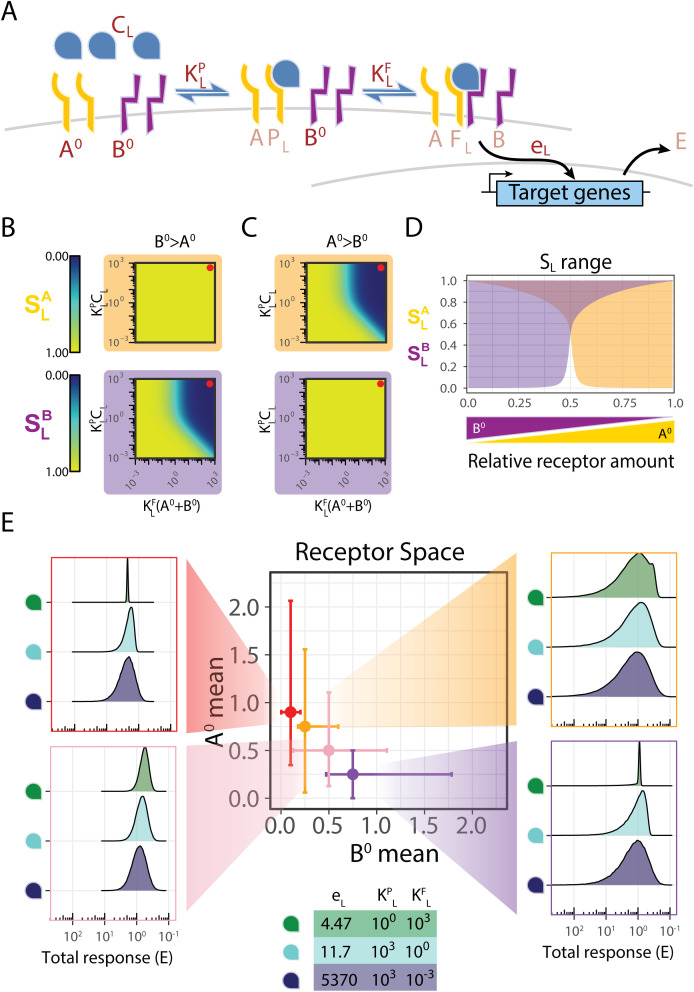
Heterodimeric receptor architecture allows for arbitrary heterogeneity-based ligand discrimination. (A) A minimal model for a pathway with a sequentially formed heterodimeric receptor. Receptor subunits and ligands bind sequentially to form partial and full complexes and activate target genes. The six model parameters are shown in dark brown, and the five variables are shown in light brown. Parameters that depend on the specific ligand identity are denoted with a subscript L. (B, C) The scaling of the model in the levels of each receptor subunits were computed across parameters for the case of *A*^*0*^ < *B*^*0*^ (B) or *A*^*0*^ > *B*^*0*^ (C). The scaling with the A subunit (*S*^*A*^_*L*_) is plotted with an orange background, while the scaling with the B subunit (*S*^*B*^_*L*_) is plotted with a purple background. The red circles indicate parameter regimes discussed in S4A, S4B Fig. (D) The ranges of the scaling levels (*S*^*A*^_*L*_ in orange and *S*^*B*^_*L*_ in purple) achievable across ligand parameters are plotted for different relative levels of the receptor subunits. (E) Heterodimeric receptor systems allow ligand identity to tune response heterogeneity independently of mean activity. This panel demonstrates the central prediction of our model. We consider four cell populations (red, yellow, pink, purple) of 100,000 cells with a Gamma distribution of receptor subunits with different means and standard deviations. The populations have different means and standard deviations for *A*^*0*^ and *B*^*0*^, which are plotted in the central panel. We simulated the response of each population to three ligands with different affinity values (bottom). For each ligand, the activity rate was set so that the population mean response would be 1. When the higher expressed subunit has lower variability across the population (red, purple), we find stronger dependence of the response heterogeneity on the ligand identity.

Dimerization of receptors does not always occur with two identical receptor subunits. In fact, many pathways, such as the BMP, TGFβ, and type I IFN pathways, utilize two types of receptor subunits to form an inherently heterodimeric complex (Table 1) [4,5,38]. We tested whether such architectures could lead to a more substantial control over the response heterogeneity compared with the two-fold limit arising in the homodimeric model. To explore this case, we added a second receptor subunit *B,* into our model (Fig 5A). Briefly, a ligand, denoted by *L*, first binds to a specific receptor subunit, denoted by *A,* to form a partial complex, *P*_*L*_, with an affinity of *K*^*P*^_*L*_. Only once *P*_*L*_ is formed it then binds the second subunit, denoted by *B*, to form the full trimeric complex, *F*_*L*_, with an affinity *K*^*F*^_*L*_. As with the previous models, once the full complex *F*_*L*_ is formed, it will induce the expression of target genes, *E*_*L*_, in a ligand-dependent activity rate, *e*_*L*_. This model represents the current knowledge for the type I IFN, and TGFβ pathways, which canonically have only one pair of receptors, and the affinity hierarchy results in a sequential complex formation [3–5,38]. Importantly, while it is sometimes possible for a ligand to bind either of the two subunits first *in-vitro* [50,51], when testing *in-vivo,* the ligand binds preferentially to one subunit, resulting in an effective sequential assembly of the full receptor [4]. Allowing the ligand to bind either receptor subunit first does not change the main conclusions (S1 Text).

The model is described by six biochemical parameters. The ligand-dependent parameters are the concentration of the ligand, *C*_*L*_, its affinity to the free subunit *A*, *K*^*P*^_*L*_, the affinity of the partial complex *P*_*L*_ to *B*, *K*^*F*^_*L,*_ and the activity rate of the resulting complex, *e*_*L*_. The set of these four parameters together defines a specific signaling environment for the cells. In addition, the configuration of the cells is defined by the level of the receptors. Here, we will parameterize this configuration using the total receptor subunits and their ratio, A^0^/B^0^. As before, we assume that ligands concentration is not significantly affected by binding to the receptors. We start by considering the case *e*_*L*_ = 1 for all ligands and solving the model in a steady state. Using dimensional analysis, we find that the system’s response depends on three-parameter combinations (S3A Fig, S1 Text).

In order to determine the capacity of ligand identity to determine response heterogeneity, we computed the scaling for this model by considering changes in each of the two receptor subunits, A and B, independently (S3A-S3D Fig, S1 Text). We denote by S^A^ the scaling associated with changes in the A subunit, and by S^B^ the scaling associated with changes in the B subunit. We find that the scaling can vary with the ligand’s biochemical parameters, indicating a tunable heterogeneity in the response, as in the homodimeric model. However, in the regime where there is an imbalance between the expression levels of the two subunits, the scaling with respect to the abundant subunit can become arbitrarily low, since adding more of it does not increase full complex formation (Figs 5B-5D, S3E). Thus S^A^ and S^B^ can take values in the range from 0 to 1. This reflects a capacity to tune the heterogeneity of the response to arbitrarily low values, in contrast to the two-fold reduction limit found for the homodimeric model. These results hold independently of the total number of subunits on the cell surface and on the values of the activity rate parameters, *e*_*L*_ (S3F Fig, S1 Text). These results also hold in a more general case where the ligand can bind either the A or B subunit first, before binding the other subunit to form a full complex (non-sequential binding model, S3G Fig). As in the case of homodimeric receptor architectures, we find that S^A^ and S^B^, are functionally dependent directly on F_L_ (S3E Fig). Overall, we find that an architecture of heterodimeric receptors enables ligands to tune significantly the scaling parameters.

Using the model, we can get intuition about the molecular mechanism allowing two distinct receptors to have more flexibility in controlling the response variability. We can consider a parameter regime where both partial-complex and full-complex affinities are low (*K*^*F*^_*L*_*(A*^*0*^ + *B*^*0*^)*<<1, K*^*P*^_*L*_*C*_*L*_*<<1*), and thus, both complexes *P*_*L*_ and *F*_*L*_ are sub-saturated. In this regime, the number of complexes depends linearly on the level of each receptor subunit. We note that this is analogous to the regime with quadratic dependence in the previous model. To see how the scaling changes, we compare this to a second regime where both affinities are high (*K*^*F*^_*L*_*(A*^*0*^ + *B*^*0*^)*>>1, K*^*P*^_*L*_*C*_*L*_*>>1*), and thus all the binding reactions are saturated (Fig 5B, 5C, red circles). In this case, all receptors form complexes as long as there are enough free receptor subunits, and the scaling with respect to a particular subunit depends on its relative abundance. In the case where *B* is the more abundant (S4A Fig), both *A* and *P*_*L*_ are saturated. Thus, the ligand *L* will directly bind all free *A* subunits to form *P*_*L*_, which will then bind to *B* to form the full complex. Accordingly, the response will be sensitive to changes in the amount of *A* (S4A Fig, right) as the limiting subunit. In contrast, as there are many more *B* subunits than *A*, most of them would remain free, not affecting the amount of *F*_*L*_ and making the response insensitive to changes in B (S4A Fig, left). When *A* is more abundant (S4B Fig), *P*_*L*_ partial complexes will form, but most of them will not be bound by B. Accordingly, while changes to *A* would affect the amount of *P*_*L*_, it wouldn’t affect the amount of *F*_*L*_, and the response would be insensitive to these changes (S4B Fig, right). However, any addition of *B* would directly bind to *P*_*L*_, increasing the amount of *F*_*L*_ (S4B Fig, left). As such, the response in this regime is sensitive to changes in *B*. This regime extends the regime with linear dependence in the homodimeric model. For intermediate parameters, the dependence of the response on the abundant subunit will be sublinear. Overall, the distinct identity of the two subunits results in a sublinear dependence of the response on each receptor, which provides for the extended flexibility in tuning the scaling metric.

### Response heterogeneity shows a larger tunability range when the expression of limiting receptors varies across cells

In order to determine how the extended range of the scaling metric affects the overall variability in the response of cell populations, we simulate the response across a cell population, as described before. However, as opposed to the previous receptor-ligand architectures, where receptors comprise a single protein variant, here there are two receptor subunits. Thus, we performed several simulations of 100,000 cells for distinct population mean expression levels for the subunit receptors *A* and *B* and for distinct levels of expression variability (Fig 5E). For each population of cells, we analyzed the response distribution under the induction of three different ligands with different parameters. As with the previous model, in order to focus on the heterogeneity of the response, we adjusted the parameters such that the population mean response is identical across ligands. We calculated the coefficient of variation for each cell population and found that the response heterogeneity varies within a large range across the distinct simulated populations. This effect is observed when there is a large variation in the expression of the two receptors and when the more abundant receptor shows larger heterogeneity across the population of cells. For these parameters, we find more than an order of magnitude difference in the response heterogeneity between ligands. When the receptor expression levels are outside this regime, the difference in heterogeneity induced by the ligands converges back to a twofold difference. Indeed, our results for the specificity show a stronger dependence on ligand parameters when the ratio of the abundances of the two receptor subunits is away from one. More specifically, the scaling relative to the more abundant receptor is tunable, and variability in that receptor will contribute to a ligand-dependent variation. In contrast, the scaling with respect to the less abundant receptor is close to 1, and large variability in that receptor will give a ligand-independent contribution to the response variability. Thus, we see that the response heterogeneity in the simulation reveals a behavior consistent with the analytical behavior of the scaling, allowing for large differences in the heterogeneity generated by different ligands.

## Discussion

Variability in biological systems has been shown to play a significant and functional role [17,18,20,24]. Thus, externally regulating the heterogeneity within a population of cells can have an important effect on biological processes [52,53]. While extracellular signals are known to control the level of intracellular activity through the signaling pathways, effects that could control the heterogeneity of the response across cells have not been studied extensively. Here we show that for specific architectures of receptor-ligand interactions, the response heterogeneity can be directly controlled by the specific ligand, with different ligands acting through the same pathway resulting in the same mean activity across the population but with different degrees of heterogeneity.

**Fig 6:**
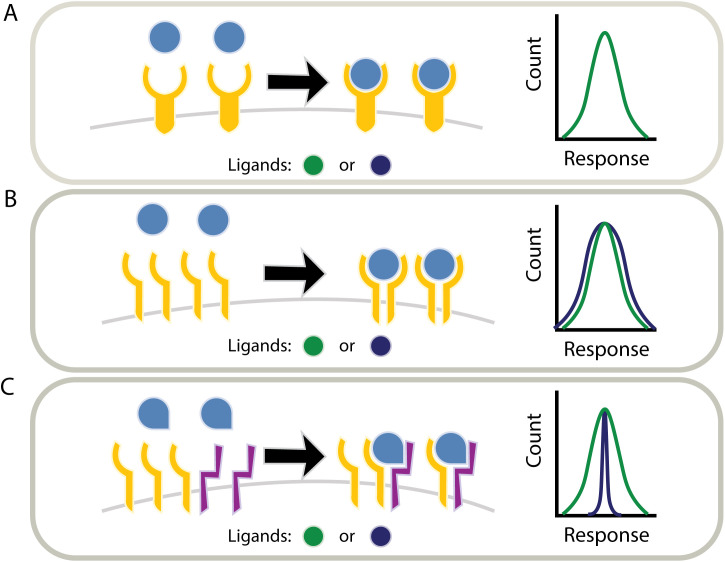
Different receptor architectures allow for different degrees of ligand discrimination based on response heterogeneity. (A) Receptors with a single-unit architecture always generate the same distribution of responses for a given mean response. Ligand identity can only affect the mean response but not the heterogeneity across the population. (B) The heterogeneity of the response for pathways containing homodimeric receptors can change by up to 2-fold, depending on the parameters of the ligands. In this way, ligands can be discriminated at the population level based on response heterogeneity. (C) Finally, pathways with heterodimeric receptors provide higher control over the response heterogeneity when the abundance of the two receptor subunits differ. This allows for a high degree of ligand discrimination for systems with an unbalanced expression of receptors.

We employed mathematical modeling to quantify the dependence of the response heterogeneity on ligand-dependent parameters. A new local scaling metric that quantifies response susceptibility to changes in pathway components enabled us to perform a local analysis of the variability in the response without assuming any specific form of the overall heterogeneity in the component. We found that pathways based on single unit receptors provide a robust and parameter-independent heterogeneity in steady-state. Thus, any two ligands, at concentrations that activate the pathway to the same mean level, result in the same population level variability as well (Fig 6A). Extending our analysis to additional receptor architectures, we found that signaling pathways initiated by receptor dimerization can result in ligand-dependent heterogeneity (Table 1). In these cases, two ligands can produce the same mean pathway activity while having distinct population heterogeneity. In particular, systems with homodimeric receptors allow for only limited differences in the heterogeneity of up to a two-fold difference in the population standard deviation (Fig 6B). However, systems with heterodimeric receptors allow for a larger fold range of heterogeneity levels when one of the receptor subunits is highly expressed and more variable (Fig 6C). Significantly, we find that for dimeric receptors, the response heterogeneity can be attenuated in a ligand-dependent manner providing a novel approach for ligand discrimination at the population level rather than by any individual cells.

In addition to ligand-dependent regulation, our analysis shows that competitive inhibitors provide a mechanism to tune response heterogeneity independently of mean activity levels. By modulating the effective efficiency, inhibitors can modify the scaling relationship between receptor abundance and complex formation, thereby tuning response heterogeneity across the population even when the overall number of signaling complexes is unchanged. This suggests a functional role for natural antagonists or synthetic inhibitors not only in dampening signaling but also in shaping the distribution of responses within a tissue. Such a mechanism could provide cells with an additional means of fine-tuning collective behaviors and may be relevant in both immune regulation and therapeutic interventions.

A general conundrum is why different biological pathways, that all similarly enable intracellular activation based on extracellular signals, utilize distinct signaling complex architectures (Table 1). Our results provide a possible functional implication for multi-subunit receptor complexes that are often used in mammalian signaling pathways. A recent study [10] demonstrated that heterodimers might provide optimal ligand discrimination through distinct activation strength. Our work suggests a distinct possible functional advantage for such pathway interaction motifs, as they enable ligand-tunable variability. Under this hypothesis, when ligands are applied at a tissue level, such as interferon, TGFβ, BMP, or other cytokines, the responses are induced across the entire population of cells. In this case, controlling the heterogeneity of the response can provide a biological advantage, and the pathway would evolve to utilize multiple heterodimeric receptors. On the other hand, if the signals are inherently operating on individual cells, these pathways could utilize a single protein receptor. For example, in the juxtacrine NOTCH pathway, ligands are directed toward a single neighboring cell only. In agreement with our hypothesis, the Notch pathway has a single-unit receptor, and ligand discrimination in this pathway is achieved through ligand-distinct temporal dynamics [8]. Overall, we find that the choice of receptor type may depend on whether the pathway functions on a whole tissue or on individual cells locally.

The type I IFN pathway, which induces an innate immune response in tissues attacked by viruses [3], is a hallmark example of a multi-ligand pathway comprising, in humans, 16 ligand variants that are secreted to activate cell populations. Ligand discrimination by individual cells in the IFN pathway has been studied extensively [6,10,54]. However, many of the features of our model are exhibited by the IFN pathway suggesting a possible role for ligands in controlling the response heterogeneity. IFN receptor is composed of two subunits that promiscuously bind the 16 ligands [4,54]. Furthermore, previous studies have demonstrated that heterogeneity in response to the type I IFN is the result of heterogeneity in the receptor subunits’ expression levels throughout the cellular population, with the two subunits being expressed at distinct levels [14,29,55]. Moreover, different cell types expressing different ratios of subunits have been shown to have differences in heterogeneity in the response to the same type I IFN ligand in a dose-dependent manner [56]. These findings suggest that the IFN pathway is in a regime that gives rise to ligand-dependent heterogeneity. Indeed, recent studies have shown, in both the type I IFN pathway and other innate immunity-related cytokines [44,56], that heterogeneity in the response might have a functional role in controlling inflammation [57,58]. As such, It would be interesting to test whether the spectrum of diverse type I IFN ligands results in distinct variability, adding to the control over the functional heterogeneity, which seems to play a major part in the immune response.

These findings raise the question of how such heterogeneity is read out downstream. Our analysis predicts that heterodimeric architectures allow ligands to tune population-level heterogeneity without necessarily altering the mean response. Such heterogeneity can have functional consequences when downstream signaling involves threshold-like behaviors, such as transcriptional activation, fate decisions, or antiviral states. Indeed, it has been demonstrated that in some pathways, graded receptor activity induces a binary transcriptional response and that such a response is regulated at the population level [59]. This has also been shown in the context of noisy thresholds [60]. In such cases, binary single-cell responses can have different population-level effects. Under stimulation that induces a heterogeneous response, the percentage of transcriptionally active cells within the population will gradually increase, producing a graded population-level output. In contrast, stimulation that induces a homogeneous response will exhibit a switch-like behavior at the population level. In this way, ligand-dependent heterogeneity described in this paper could allow cells to control their activation mode and influence collective outcomes.

Finally, cells in a population generally sense combinations of ligands within the same pathway interacting promiscuously with the receptors [5,15,16]. In particular, type I IFN is known to have 16 ligands that can all signal through the same set of receptors [6,54]. More generally, many pathways beyond IFN are activated by multiple redundant ligands, including the TGFb pathway, activated by 16 ligand variants, the BMP pathway, activated by 14 ligands, the Wnt pathway, activated by 19 ligands, and the FGF pathway, activated by 18 ligands. It would be interesting to extend our model to such cases of ligand combinations. In particular, using combinations of a high-variability inducing ligand with a low-variability inducing ligand might provide a way to regulate the heterogeneity of the response continuously, as suggested by the results for the ALA model.

Such experimental systems, in which multiple ligands share the same receptor set, also provide a context where our model makes testable predictions. For example, our analysis predicts that in the IFN system, certain ligand variants will produce distinct levels of population-level response heterogeneity even at matched mean activity levels. In particular, ligands with different enzymatic activities are expected to induce distinct degrees of heterogeneity despite the same average response. Such differences could be directly measurable by flow cytometry or single-cell RNA-seq by quantifying the cell-to-cell variance in downstream signaling markers. The activity level of individual ligands could be assessed by the saturation response to each ligand. Importantly, our analysis also predicts that adding competitive inhibitors should attenuate the heterogeneity of the response. Similar tests could be envisioned for TGF-β/BMP or FGF ligands that share receptors but differ in binding parameters. These experiments would provide a direct way to evaluate how heterodimerization enables ligand-dependent tuning of population-level heterogeneity.

Ligands mediate information between cells to generate robust, coordinated responses. Mostly, ligands are considered to act on individual cells and regulate their level of activity. We have shown that ligands can further determine population-level properties, such as the heterogeneity of the population independently of the average response level. Furthering our understanding of the capacity of ligands to regulate population-level features will bring us closer to understanding major biological processes such as the immune response, cancer, autoimmune diseases, and multicellular development. Moreover, a better understanding of controlling and manipulating heterogeneity within a population of cells would have implications in synthetic biology and artificial circuit design.

## Methods

We have developed analytical mathematical models for the different ligand-receptor architectures using binding-unbinding kinetic equations. Models were solved for the steady state response analytically, and the solutions were validated using the Equilibrium Toolkit (EQTK). EQTK is an optimized Python-based numerical solver for biochemical reaction systems [48,49]. Parameter scanning and simulations were performed using R version 3.6.3. Further details are provided in the S1 Text.

## Supporting information

S1 Fig Single-unit receptor response and scaling. (A) We consider The formation of *F*_*L*_ for the single unit receptor pathway given the five different ligands described in Fig 1B over multiple concentrations and *A*^*0*^* = 1*. (B) The dependence of the response on the receptors is plotted for two systems with either high (blue) or low (green) scaling. Given a specific distribution of receptors in a population of cells, the high-sensitivity system (blue) with larger scaling will generate a highly variable response. In contrast, the low-sensitivity system (green) has lower scaling and will generate responses with lower variability. ARU = Arbitrary Receptor Units, ACU = Arbitrary Concentration Units.
(TIF)

S2 Fig Homodimeric receptor response heterogeneity varies and correlates with its scaling to subunit abundance. (A) The homodimeric model’s (ALA) full complex (*F*_*L*_) is plotted across model parameters, showing a non-monotonic response to ligand concentrations. (B) A minimal model for a pathway with a homodimeric receptor with two ligands (LAAL model). A single ligand molecule binds sequentially to a receptor subunit to form a partial (P) complex. Two partial complexes bind to form a full complex (F) and activate target genes. The five model parameters are shown in dark brown, while the four variables are shown in light brown. Parameters that depend on the specific ligand identity are denoted with a subscript L. (C) The LAAL model’s full complex (FL) is plotted across model parameters. (D) The LAAL model’s scaling (S) to changes in the receptor subunit A0 is plotted as model parameters vary. (E) The standard deviation in the response was calculated for the ALA model (cf. Fig 4C) and plotted for ligands with different scaling values. Different ligands are colored as in Fig 4B. The relationship can be approximated by a linear dependence (rho = 0.997, p = 0.0001683, Pearson correlation).
(TIF)

S3 Fig Heterodimeric receptor architecture extends the ligand-dependent control of response heterogeneity. (A - C) The sequential binding heterodimeric receptor model’s full complex (*F*_*L*_) (A) and the scaling with receptor subunits *A*^*0*^ and *B*^*0*^ (B and C, respectively) are shown across the model’s dimensionless parameters as discussed in Fig 5B,5C and across five different ratios of the receptor subunits *A*^*0*^/*B*^*0*^ [0.001, 0.25, 0.5, 0.75, 999]. (D) Total scaling is determined by the addition of the scaling with *A*^*0*^ and *B*^*0*^ as shown in B and C. (E) The scaling with each subunit is determined by its given relative amount and the fraction of bound receptors (*F*_*L*_/[the less abundant subunit]). This dependence is plotted for different ratios of subunits *A*^*0*^ and *B*^*0*^. (F) Range of the model’s scaling with the receptor subunits (*S*^*A*^_*L*_ in orange and *S*^*B*^_*L*_ in purple), given different total amounts of the receptor subunits (shades of orange and purple). The range was calculated under the same model parameters and subunit ratios as in Fig 5D. (G) Range of the scaling parameters (*S*^*A*^_*L*_ in orange and *S*^*B*^_*L*_ in purple) for a model with non-sequential subunit binding (see Supplementary Information). The scaling parameters retain a similar range to the sequential model (cf. S3G Fig).
(TIF)

S4 Fig Cartoon representing the molecular mechanism behind the dependence of the scaling on the relative receptor amounts in the high-affinity regime. (A) When *B*^*0*^ is larger than *A*^*0,*^ and affinities are high (ligand concentrations are saturating, red circles in Fig 5B), all free ligands will bind directly to *A* (yellow), leaving no free subunit, and all *B* will bind to any free *P*_*L*_. As *A*^*0*^ > *B*^*0*^, there are more free *B* than free *P*_*L*_, making the complex amount *F*_*L*_ insensitive to *B* and dependent on *A*. (B) Alternatively, when *A*^*0*^ is more abundant than *B*^*0*^, all B subunits will form a full complex, *F*_*L*_. In this case, *F*_*L*_ is strongly dependent on *B* and insensitive to *P*_*L*_ and, thus, to *A*.
(TIF)

S5 Fig Sensitivity of models with non-constant ligand concentration. (A) The sensitivity, S_L_, was calculated for a model with receptor architecture of a single receptor subunit (AL), assuming small number of ligand molecules. The calculations were done for different initial receptor (A^0^) and ligand (C^0^_L_) amounts, as well as different ligand-receptor affinities (K_L_). (B) Numeric simulations of the sensitivity, S_L_, were done for the ALB model assuming small number of ligands. The range of the model’s scaling with the receptor subunits (S^A^_L_ in orange and S^B^_L_ in purple), given different total amounts of the receptor subunits (shades of orange and purple).
(TIF)

S1 Text Supplementary Information. (PDF)

## References

[1] Antebi YE, Nandagopal N, Elowitz MB. An operational view of intercellular signaling pathways. Curr Opin Syst Biol. 2017;1:16–24. doi: 10.1016/j.coisb.2016.12.003 29104946 PMC5665397

[2] Rogers KW, Schier AF. Morphogen gradients: from generation to interpretation. Annu Rev Cell Dev Biol. 2011;27:377–407. doi: 10.1146/annurev-cellbio-092910-154148 21801015

[3] Ivashkiv LB, Donlin LT. Regulation of type I interferon responses. Nat Rev Immunol. 2014;14(1):36–49. doi: 10.1038/nri3581 24362405 PMC4084561

[4] Schreiber G, Piehler J. The molecular basis for functional plasticity in type I interferon signaling. Trends Immunol. 2015;36(3):139–49. doi: 10.1016/j.it.2015.01.002 25687684

[5] Antebi YE, Linton JM, Klumpe H, Bintu B, Gong M, Su C, et al. Combinatorial Signal Perception in the BMP Pathway. Cell. 2017;170(6):1184-1196.e24. doi: 10.1016/j.cell.2017.08.015 28886385 PMC5612783

[6] Fathi S, Nayak CR, Feld JJ, Zilman AG. Absolute Ligand Discrimination by Dimeric Signaling Receptors. Biophys J. 2016;111(5):917–20. doi: 10.1016/j.bpj.2016.07.029 27602720 PMC5018129

[7] Andrews MG, Del Castillo LM, Ochoa-Bolton E, Yamauchi K, Smogorzewski J, Butler SJ. BMPs direct sensory interneuron identity in the developing spinal cord using signal-specific not morphogenic activities. Elife. 2017;6:e30647. doi: 10.7554/eLife.30647 28925352 PMC5605194

[8] Nandagopal N, Santat LA, LeBon L, Sprinzak D, Bronner ME, Elowitz MB. Dynamic Ligand Discrimination in the Notch Signaling Pathway. Cell. 2018;172(4):869-880.e19. doi: 10.1016/j.cell.2018.01.002 29398116 PMC6414217

[9] Adelaja A, Taylor B, Sheu KM, Liu Y, Luecke S, Hoffmann A. Six distinct NFκB signaling codons convey discrete information to distinguish stimuli and enable appropriate macrophage responses. Immunity. 2021;54(5):916-930.e7. doi: 10.1016/j.immuni.2021.04.011 33979588 PMC8184127

[10] Binder P, Schnellbächer ND, Höfer T, Becker NB, Schwarz US. Optimal ligand discrimination by asymmetric dimerization and turnover of interferon receptors. Proc Natl Acad Sci U S A. 2021;118(37):e2103939118. doi: 10.1073/pnas.2103939118 34507994 PMC8449373

[11] Kirby D, Parmar B, Fathi S, Marwah S, Nayak CR, Cherepanov V, et al. Determinants of Ligand Specificity and Functional Plasticity in Type I Interferon Signaling. Front Immunol. 2021;12:748423. doi: 10.3389/fimmu.2021.748423 34691060 PMC8529159

[12] Piehler J, Thomas C, Garcia KC, Schreiber G. Structural and dynamic determinants of type I interferon receptor assembly and their functional interpretation. Immunol Rev. 2012;250(1):317–34. doi: 10.1111/imr.12001 23046138 PMC3986811

[13] Jaitin DA, Roisman LC, Jaks E, Gavutis M, Piehler J, Van der Heyden J, et al. Inquiring into the differential action of interferons (IFNs): an IFN-alpha2 mutant with enhanced affinity to IFNAR1 is functionally similar to IFN-beta. Mol Cell Biol. 2006;26(5):1888–97. doi: 10.1128/MCB.26.5.1888-1897.2006 16479007 PMC1430259

[14] Levin D, Harari D, Schreiber G. Stochastic receptor expression determines cell fate upon interferon treatment. Mol Cell Biol. 2011;31(16):3252–66. doi: 10.1128/MCB.05251-11 21690295 PMC3147786

[15] Klumpe HE, Langley MA, Linton JM, Su CJ, Antebi YE, Elowitz MB. The context-dependent, combinatorial logic of BMP signaling. Cell Syst. 2022;13(5):388-407.e10. doi: 10.1016/j.cels.2022.03.002 35421361 PMC9127470

[16] Su CJ, Murugan A, Linton JM, Yeluri A, Bois J, Klumpe H, et al. Ligand-receptor promiscuity enables cellular addressing. Cell Syst. 2022;13(5):408-425.e12. doi: 10.1016/j.cels.2022.03.001 35421362 PMC10897978

[17] Eldar A, Elowitz MB. Functional roles for noise in genetic circuits. Nature. 2010;467(7312):167–73. doi: 10.1038/nature09326 20829787 PMC4100692

[18] Losick R, Desplan C. Stochasticity and cell fate. Science. 2008;320(5872):65–8. doi: 10.1126/science.1147888 18388284 PMC2605794

[19] Elowitz MB, Levine AJ, Siggia ED, Swain PS. Stochastic gene expression in a single cell. Science. 2002;297(5584):1183–6. doi: 10.1126/science.1070919 12183631

[20] Paszek P, Ryan S, Ashall L, Sillitoe K, Harper CV, Spiller DG, et al. Population robustness arising from cellular heterogeneity. Proc Natl Acad Sci U S A. 2010;107(25):11644–9. doi: 10.1073/pnas.0913798107 20534546 PMC2895068

[21] Adlung L, Stapor P, Tönsing C, Schmiester L, Schwarzmüller LE, Postawa L, et al. Cell-to-cell variability in JAK2/STAT5 pathway components and cytoplasmic volumes defines survival threshold in erythroid progenitor cells. Cell Rep. 2021;36(6):109507. doi: 10.1016/j.celrep.2021.109507 34380040

[22] Kalmar T, Lim C, Hayward P, Muñoz-Descalzo S, Nichols J, Garcia-Ojalvo J, et al. Regulated fluctuations in nanog expression mediate cell fate decisions in embryonic stem cells. PLoS Biol. 2009;7(7):e1000149. doi: 10.1371/journal.pbio.1000149 19582141 PMC2700273

[23] Yaakov G, Lerner D, Bentele K, Steinberger J, Barkai N. Coupling phenotypic persistence to DNA damage increases genetic diversity in severe stress. Nat Ecol Evol. 2017;1(1):16. doi: 10.1038/s41559-016-0016 28812556

[24] Carey JN, Mettert EL, Roggiani M, Myers KS, Kiley PJ, Goulian M. Regulated Stochasticity in a Bacterial Signaling Network Permits Tolerance to a Rapid Environmental Change. Cell. 2018;173(1):196-207.e14. doi: 10.1016/j.cell.2018.02.005 29502970 PMC5866230

[25] Kamino K, Keegstra JM, Long J, Emonet T, Shimizu TS. Adaptive tuning of cell sensory diversity without changes in gene expression. Sci Adv. 2020;6(46):eabc1087. doi: 10.1126/sciadv.abc1087 33188019 PMC7673753

[26] Shaffer SM, Dunagin MC, Torborg SR, Torre EA, Emert B, Krepler C, et al. Rare cell variability and drug-induced reprogramming as a mode of cancer drug resistance. Nature. 2017;546(7658):431–5. doi: 10.1038/nature22794 28607484 PMC5542814

[27] You S-T, Jhou Y-T, Kao C-F, Leu J-Y. Experimental evolution reveals a general role for the methyltransferase Hmt1 in noise buffering. PLoS Biol. 2019;17(10):e3000433. doi: 10.1371/journal.pbio.3000433 31613873 PMC6814240

[28] Hall JC, Rosen A. Type I interferons: crucial participants in disease amplification in autoimmunity. Nat Rev Rheumatol. 2010;6(1):40–9. doi: 10.1038/nrrheum.2009.237 20046205 PMC3622245

[29] Shalek AK, Satija R, Shuga J, Trombetta JJ, Gennert D, Lu D, et al. Single-cell RNA-seq reveals dynamic paracrine control of cellular variation. Nature. 2014;510(7505):363–9. doi: 10.1038/nature13437 24919153 PMC4193940

[30] Steen A, Larsen O, Thiele S, Rosenkilde MM. Biased and g protein-independent signaling of chemokine receptors. Front Immunol. 2014;5:277. doi: 10.3389/fimmu.2014.00277 25002861 PMC4066200

[31] Vanamee ÉS, Faustman DL. Structural principles of tumor necrosis factor superfamily signaling. Sci Signal. 2018;11(511):eaao4910. doi: 10.1126/scisignal.aao4910 29295955

[32] Lemmon MA, Schlessinger J. Cell signaling by receptor tyrosine kinases. Cell. 2010;141(7):1117–34. doi: 10.1016/j.cell.2010.06.011 20602996 PMC2914105

[33] Ornitz DM, Itoh N. The Fibroblast Growth Factor signaling pathway. Wiley Interdiscip Rev Dev Biol. 2015;4(3):215–66. doi: 10.1002/wdev.176 25772309 PMC4393358

[34] Abud HE, Chan WH, Jardé T. Source and Impact of the EGF Family of Ligands on Intestinal Stem Cells. Front Cell Dev Biol. 2021;9:685665. doi: 10.3389/fcell.2021.685665 34350179 PMC8327171

[35] Wack A, Terczyńska-Dyla E, Hartmann R. Guarding the frontiers: the biology of type III interferons. Nat Immunol. 2015;16(8):802–9. doi: 10.1038/ni.3212 26194286 PMC7096991

[36] Green DS, Young HA, Valencia JC. Current prospects of type II interferon γ signaling and autoimmunity. J Biol Chem. 2017;292(34):13925–33. doi: 10.1074/jbc.R116.774745 28652404 PMC5572907

[37] Kim Y-M, Shin E-C. Type I and III interferon responses in SARS-CoV-2 infection. Exp Mol Med. 2021;53(5):750–60. doi: 10.1038/s12276-021-00592-0 33953323 PMC8099704

[38] Vilar JMG, Jansen R, Sander C. Signal processing in the TGF-beta superfamily ligand-receptor network. PLoS Comput Biol. 2006;2(1):e3. doi: 10.1371/journal.pcbi.0020003 16446785 PMC1356091

[39] Akdis M, Aab A, Altunbulakli C, Azkur K, Costa RA, Crameri R, et al. Interleukins (from IL-1 to IL-38), interferons, transforming growth factor β, and TNF-α: Receptors, functions, and roles in diseases. J Allergy Clin Immunol. 2016;138(4):984–1010. doi: 10.1016/j.jaci.2016.06.033 27577879

[40] Chen Y-G. Endocytic regulation of TGF-beta signaling. Cell Res. 2009;19(1):58–70. doi: 10.1038/cr.2008.315 19050695

[41] Di Guglielmo GM, Le Roy C, Goodfellow AF, Wrana JL. Distinct endocytic pathways regulate TGF-beta receptor signalling and turnover. Nat Cell Biol. 2003;5(5):410–21. doi: 10.1038/ncb975 12717440

[42] Zheng H, Qian J, Baker DP, Fuchs SY. Tyrosine phosphorylation of protein kinase D2 mediates ligand-inducible elimination of the Type 1 interferon receptor. J Biol Chem. 2011;286(41):35733–41. doi: 10.1074/jbc.M111.263608 21865166 PMC3195636

[43] Kumar KG, Suresh K. SCFHOS ubiquitin ligase mediates the ligand-induced down-regulation of the interferon- receptor. The EMBO Journal. 2003;22:5480–90. 14532120 10.1093/emboj/cdg524 PMC213778

[44] Topolewski P, Zakrzewska KE, Walczak J, Nienałtowski K, Müller-Newen G, Singh A, et al. Phenotypic variability, not noise, accounts for most of the cell-to-cell heterogeneity in IFN-γ and oncostatin M signaling responses. Sci Signal. 2022;15(721):eabd9303. doi: 10.1126/scisignal.abd9303 35167339

[45] Moraga I, Harari D, Schreiber G, Uzé G, Pellegrini S. Receptor density is key to the alpha2/beta interferon differential activities. Mol Cell Biol. 2009;29(17):4778–87. doi: 10.1128/MCB.01808-08 19564411 PMC2725717

[46] Whitty A, Borysenko CW. Small molecule cytokine mimetics. Chem Biol. 1999;6(4):R107-18. doi: 10.1016/S1074-5521(99)80034-9 10099129

[47] Perelson AS, DeLisi C. Receptor clustering on a cell surface. I. Theory of receptor cross-linking by ligands bearing two chemically identical functional groups. Math Biosci. 1980;48:71–110.

[48] Dirks RM, Bois JS, Schaeffer JM, Winfree E, Pierce NA. Thermodynamic Analysis of Interacting Nucleic Acid Strands. SIAM Rev. 2007;49(1):65–88. doi: 10.1137/060651100

[49] Bois JS. Justin bois/eqtk: Version 0.1.1. 2020. doi: 10.22002/D1.1430

[50] Jaks E, Gavutis M, Uzé G, Martal J, Piehler J. Differential receptor subunit affinities of type I interferons govern differential signal activation. J Mol Biol. 2007;366(2):525–39. doi: 10.1016/j.jmb.2006.11.053 17174979

[51] Roder F, Wilmes S, Richter CP, Piehler J. Rapid transfer of transmembrane proteins for single molecule dimerization assays in polymer-supported membranes. ACS Chem Biol. 2014;9(11):2479–84. doi: 10.1021/cb5005806 25203456

[52] Feinerman O, Veiga J, Dorfman JR, Germain RN, Altan-Bonnet G. Variability and robustness in T cell activation from regulated heterogeneity in protein levels. Science. 2008;321(5892):1081–4. doi: 10.1126/science.1158013 18719282 PMC2673522

[53] Jeschke M, Baumgärtner S, Legewie S. Determinants of cell-to-cell variability in protein kinase signaling. PLoS Comput Biol. 2013;9(12):e1003357. doi: 10.1371/journal.pcbi.1003357 24339758 PMC3854479

[54] Thomas C, Moraga I, Levin D, Krutzik PO, Podoplelova Y, Trejo A, et al. Structural linkage between ligand discrimination and receptor activation by type I interferons. Cell. 2011;146(4):621–32. doi: 10.1016/j.cell.2011.06.048 21854986 PMC3166218

[55] Shalek AK, Satija R, Adiconis X, Gertner RS, Gaublomme JT, Raychowdhury R, et al. Single-cell transcriptomics reveals bimodality in expression and splicing in immune cells. Nature. 2013;498(7453):236–40. doi: 10.1038/nature12172 23685454 PMC3683364

[56] Nienałtowski K, Rigby RE, Walczak J, Zakrzewska KE, Głów E, Rehwinkel J, et al. Fractional response analysis reveals logarithmic cytokine responses in cellular populations. Nat Commun. 2021;12(1):4175. doi: 10.1038/s41467-021-24449-2 34234126 PMC8263596

[57] Moreau TRJ, Bondet V, Rodero MP, Duffy D. Heterogeneity and functions of the 13 IFN-α subtypes - lucky for some?. Eur J Immunol. 2023;53(8):e2250307. doi: 10.1002/eji.202250307 37367434

[58] Van Eyndhoven LC, Singh A, Tel J. Decoding the dynamics of multilayered stochastic antiviral IFN-I responses. Trends Immunol. 2021;42(9):824–39. doi: 10.1016/j.it.2021.07.004 34364820

[59] Kovary KM, Taylor B, Zhao ML, Teruel MN. Expression variation and covariation impair analog and enable binary signaling control. Mol Syst Biol. 2018;14(5):e7997. doi: 10.15252/msb.20177997 29759982 PMC5951153

[60] Vilar JMG. Noisy-threshold control of cell death. BMC Syst Biol. 2010;4:152. doi: 10.1186/1752-0509-4-152 21067567 PMC2992511

