# Supplementary figures and images for: Receptor dimerization enables ligand discrimination through tunable response heterogeneity
Source: PLoS Comput Biol. 2025 Dec 3;21(12):e1013781. doi: 10.1371/journal.pcbi.1013781 (PMC12688120; doi:10.1371/journal.pcbi.1013781)

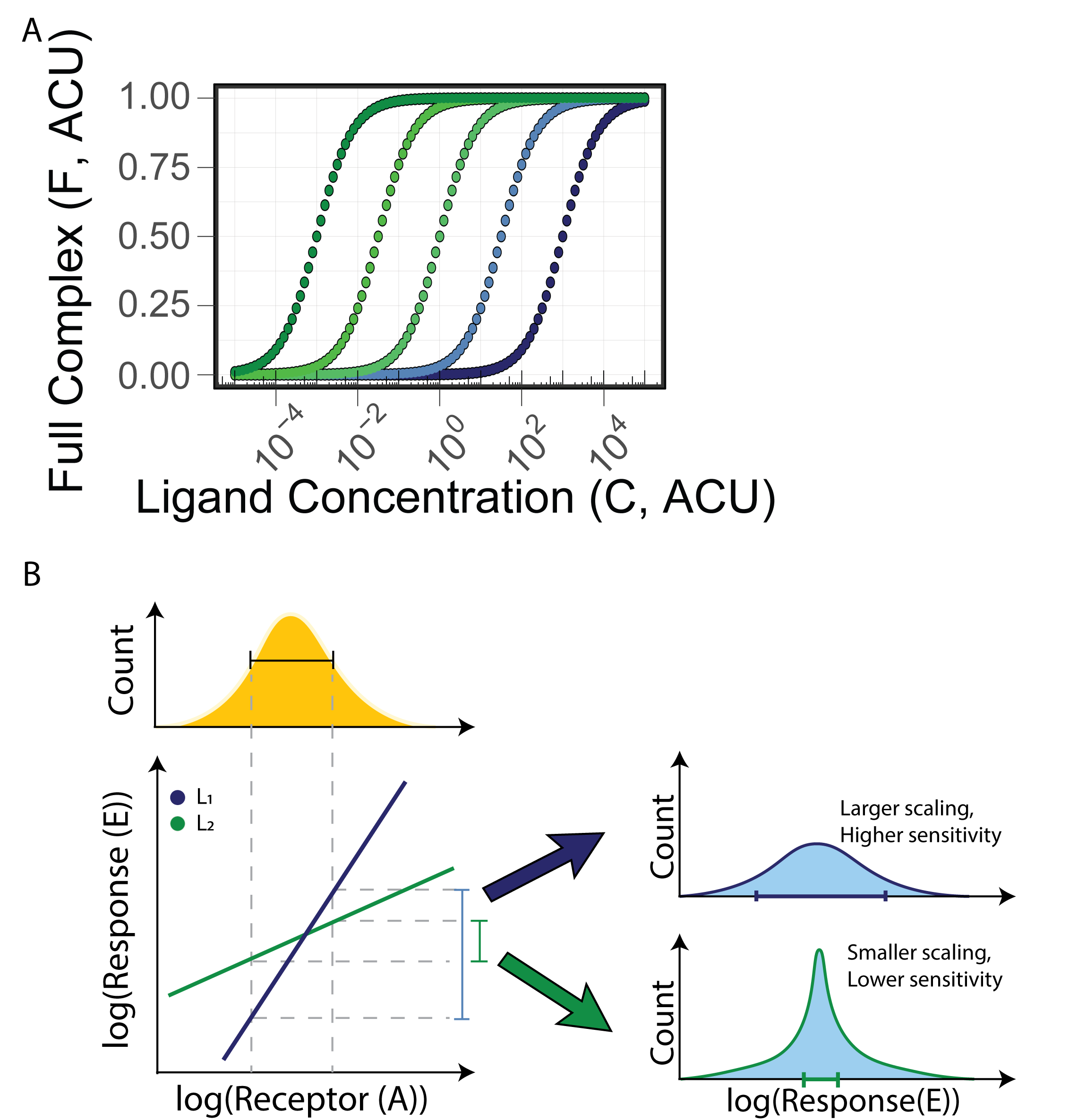

Supplement: S1 Fig — (A) We consider The formation of FL for the single unit receptor pathway given the five different ligands described in Fig 1B over multiple concentrations and A0 = 1. (B) The dependence of the response on the receptors is plotted for two systems with either high (blue) or low (green) scaling. Given a specific distribution of receptors in a population of cells, the high-sensitivity system (blue) with larger scaling will generate a highly variable response. In contrast, the low-sensitivity system (green) has lower scaling and will generate responses with lower variability. ARU = Arbitrary Receptor Units, ACU = Arbitrary Concentration Units. (TIF) [file pcbi.1013781.s001.tif]

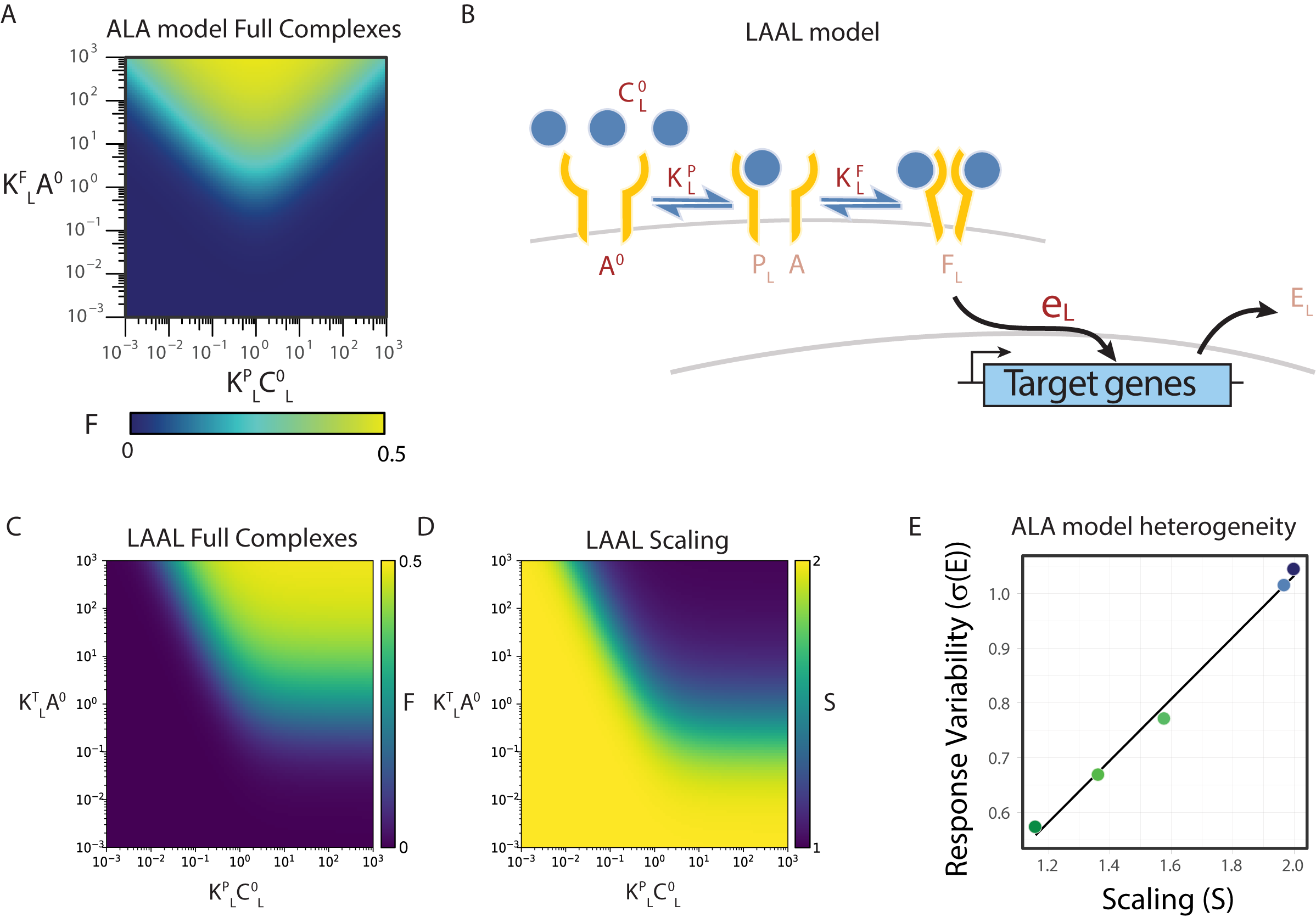

Supplement: S2 Fig — (A) The homodimeric model’s (ALA) full complex (FL) is plotted across model parameters, showing a non-monotonic response to ligand concentrations. (B) A minimal model for a pathway with a homodimeric receptor with two ligands (LAAL model). A single ligand molecule binds sequentially to a receptor subunit to form a partial (P) complex. Two partial complexes bind to form a full complex (F) and activate target genes. The five model parameters are shown in dark brown, while the four variables are shown in light brown. Parameters that depend on the specific ligand identity are denoted with a subscript L. (C) The LAAL model’s full complex (FL) is plotted across model parameters. (D) The LAAL model’s scaling (S) to changes in the receptor subunit A0 is plotted as model parameters vary. (E) The standard deviation in the response was calculated for the ALA model (cf. Fig 4C) and plotted for ligands with different scaling values. Different ligands are colored as in Fig 4B. The relationship can be approximated by a linear dependence (rho = 0.997, p = 0.0001683, Pearson correlation). (TIF) [file pcbi.1013781.s002.tif]

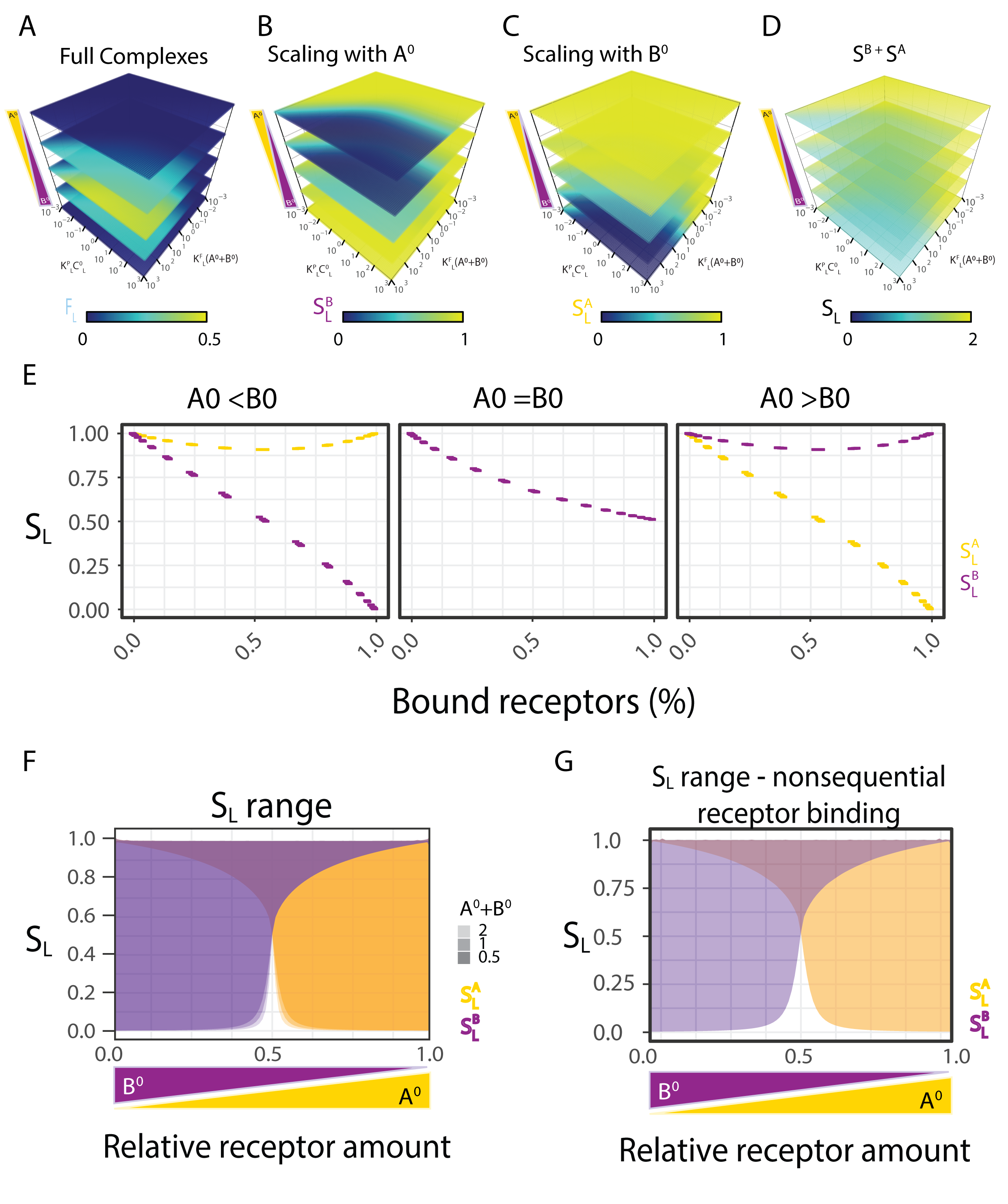

Supplement: S3 Fig — (A - C) The sequential binding heterodimeric receptor model’s full complex (FL) (A) and the scaling with receptor subunits A0 and B0 (B and C, respectively) are shown across the model’s dimensionless parameters as discussed in Fig 5B,5C and across five different ratios of the receptor subunits A0/B0 [0.001, 0.25, 0.5, 0.75, 999]. (D) Total scaling is determined by the addition of the scaling with A0 and B0 as shown in B and C. (E) The scaling with each subunit is determined by its given relative amount and the fraction of bound receptors (FL/[the less abundant subunit]). This dependence is plotted for different ratios of subunits A0 and B0. (F) Range of the model’s scaling with the receptor subunits (SAL in orange and SBL in purple), given different total amounts of the receptor subunits (shades of orange and purple). The range was calculated under the same model parameters and subunit ratios as in Fig 5D. (G) Range of the scaling parameters (SAL in orange and SBL in purple) for a model with non-sequential subunit binding (see Supplementary Information). The scaling parameters retain a similar range to the sequential model (cf. S3G Fig). (TIF) [file pcbi.1013781.s003.tif]

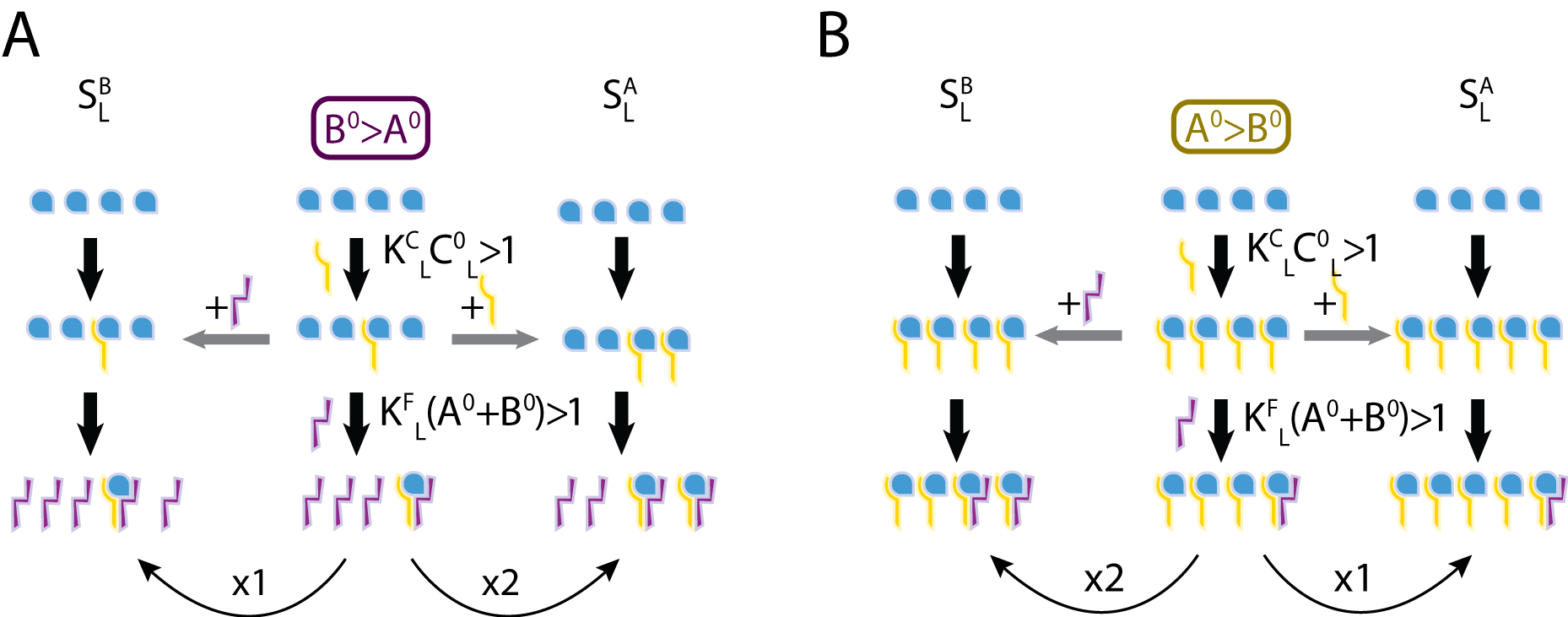

Supplement: S4 Fig — (A) When B0 is larger than A0, and affinities are high (ligand concentrations are saturating, red circles in Fig 5B), all free ligands will bind directly to A (yellow), leaving no free subunit, and all B will bind to any free PL. As A0 > B0, there are more free B than free PL, making the complex amount FL insensitive to B and dependent on A. (B) Alternatively, when A0 is more abundant than B0, all B subunits will form a full complex, FL. In this case, FL is strongly dependent on B and insensitive to PL and, thus, to A. (TIF) [file pcbi.1013781.s004.tif]

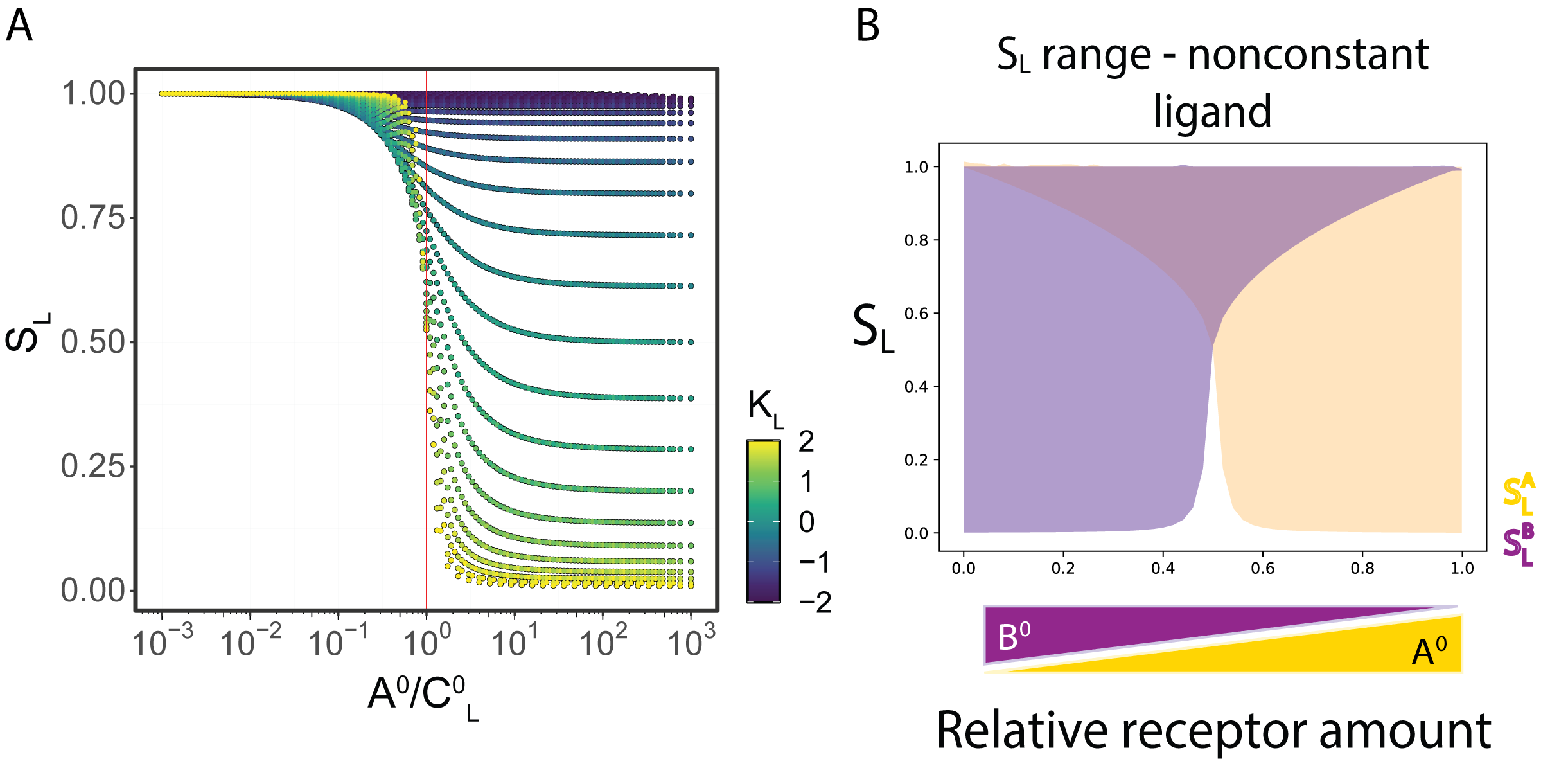

Supplement: S5 Fig — (A) The sensitivity, SL, was calculated for a model with receptor architecture of a single receptor subunit (AL), assuming small number of ligand molecules. The calculations were done for different initial receptor (A0) and ligand (C0L) amounts, as well as different ligand-receptor affinities (KL). (B) Numeric simulations of the sensitivity, SL, were done for the ALB model assuming small number of ligands. The range of the model’s scaling with the receptor subunits (SAL in orange and SBL in purple), given different total amounts of the receptor subunits (shades of orange and purple). (TIF) [file pcbi.1013781.s005.tif]
