# Supplementary material for: Receptor dimerization enables ligand discrimination through tunable response heterogeneity
Source: PLoS Comput Biol. 2025 Dec 3;21(12):e1013781. doi: 10.1371/journal.pcbi.1013781 (PMC12688120; doi:10.1371/journal.pcbi.1013781)
Supplement: S1 Text — (PDF) [file pcbi.1013781.s006.pdf]

Supplementary Information

Receptor architecture mathematical models

We set out to develop mathematical models to describe the binding of ligands and the subsequent cellular responses for three different receptor architectures. We consider a basic model with a single unit receptor (the AL model), a model with a homodimeric receptor composed of two identical subunits (the ALA model), and a model with a heterodimeric receptor composed of two different subunits (the ALB model). Specifically, we focus on the level of ligand-receptor complex formation, leading to the activation of an intracellular signal mediator. The model does not contain other processes that might affect the cellular response, such as feedback loops, non-canonical signaling, and enzymatic signal amplification.

The AL model for ligand binding by a single-unit receptor architecture

In a single-unit receptor model, which we refer to as the AL model, we consider stimulation of the pathway by the ligand *L*, at a concentration C_L. The ligand binds to a receptor, *A*, and forms a full complex F_L. We consider a first-order kinetics where the forward binding rate k_{fL} and the reverse binding rate k_{rL} are intrinsic properties of the ligand and depend on its identity. This reaction can be summarized as

$$A + L \underset{k_{rL}}{\overset{k_{fL}}{\rightleftharpoons}} F_L \quad (1)$$

In addition, for all models we assume that the volume for the ligands is large so there are significantly more ligand molecules than receptors, or $V \rightarrow \infty$. Under this assumption, ligand concentrations remain constant, such that the initial concentration, C^0_L, doesn't change and

$$C_L = C^0_L. \quad (2)$$

With this we can write the dynamical equations that describe the ligand binding by a single unit receptor:

$$\frac{dA}{dt} = k_{rL}F_L - k_{fL}AC^0_L. \quad (3)$$

Here C^0_L denotes the concentration of the ligand in volume *V*, while *A* and F_L denote the absolute number of receptors and complexes on the surface of a cell. We assume that production and consumption of the different molecules are in steady state, allowing us to neglect the endocytosis of ligands and receptors. The conservation of mass requires that the total number of each type of molecule remains constant, regardless of whether it is free or in a complex with other species. Denoting the initial receptor level as A^0 we obtain

$$A^0 = A + F_L$$

or

$$A = A^0 - F_L. \quad (4)$$

As changes in the ligand binding by receptors occur much faster than changes in receptor expression [5], we consider the behavior of the system to be at steady state. As such, all time derivatives become zero, and equation *3* can be solved to give:

$$F_L = K_L A C^0_L \quad (5)$$

where K_L is defined as $K_L \equiv \frac{k_{fL}}{k_{rL}}$, and describes the affinity of ligand L to receptor A. Plugging equation *4* into *5* we can solve the behavior of complex F_L in steady state

$$F_L = K_L C^0_L (A^0 - F_L)$$

$$F_L = \frac{K_L C^0_L}{1+K_L C^0_L} A^0. \quad (6)$$

This equation provides the dependence of the number of full complexes on the three model parameters, and simulating the parameters gives the expected Michaelis-Menten relationship (supplementary Figure 1A) [6,10].

The ALA model for ligand binding by a homodimer receptor

We next consider an architecture with two identical receptor subunits that homodimerize upon ligand binding, which we refer to as the ALA model. We consider a ligand L, at a concentration of C_L that binds to a receptor subunit, denoted by A, and forming a partial complex, P_L. This partial complex further binds a second subunit to form the full complex F_L. As before, we assume that the binding is reversible with first-order kinetics. We consider the forward and reverse binding rates for the formation of the partial complexes, k^P_{fL} and k^P_{rL}, and full complexes k^F_{fL} and k^F_{rL}, to be intrinsic properties of the specific ligand variant. These reactions can be summarized as

$$A + L \underset{k^P_{rL}}{\overset{k^P_{fL}}{\rightleftharpoons}} P_L \quad (7)$$

$$A + P_L \underset{k^F_{rL}}{\overset{k^F_{fL}}{\rightleftharpoons}} F_L \quad (8)$$

We use $C^0_L$to denote the concentration of the ligand while A, P_L and F_L denote the number of receptors and complexes on the surface of a cell. As above, we consider the concentration of the ligand to remain constant throughout the reaction (equation 2). We can now write the dynamical equations resulting from these reactions (7,8):

$$\frac{dA}{dt} = k^{P}_{rL} P_L + k^{F}_{rL} F_L - k^{P}_{fL} A C^{0}_{L} - k^{F}_{fL} A P_L \quad (9)$$

$$\frac{dP_L}{dt} = k^{P}_{fL} A C^{0}_{L} + k^{F}_{rL} F_L - k^{P}_{rL} P_L - k^{F}_{fL} A P_L \quad (10)$$

$$\frac{dF_L}{dt} = k^{F}_{fL} A P_L - k^{F}_{rL} F_L. \quad (11)$$

Considering conservation of mass, and denoting the initial values of the receptor subunits as A^0, we obtain

$$A^{0} = A + P_L + 2F_L$$

$$A = A^{0} - P_L - 2F_L. \quad (12)$$

Note that since a single complex, F_L, comprises two receptor units it appears in the conservation of mass equation with a factor of two.

The steady state solution for this model is achieved by setting equations 9, *10* and *11* to zero. Solving the resulting system of equations we find

$$P_L = K^{P}_{L} A C^{0}_{L} \quad (13)$$

$$F_L = K^{F}_{L} A P_L. \quad (14)$$

Where K^P_L is defined as $K^{P}_{L} \equiv \frac{k^{P}_{fL}}{k^{P}_{rL}}$, and K^F_L is defined as $K^{F}_{L} \equiv \frac{k^{F}_{fL}}{k^{F}_{rL}}$, denoting the affinity of the ligand and ligand-bound receptor subunit to subunit *A*. By plugging equation *12* into equations *13* and *14*, we can obtain the dependence of *P* on the parameters in steady state:

$$P_L = K^{P}_{L} C^{0}_{L} (A^{0} - P_L - 2F_L)$$

$$P_L = \frac{K^{P}_{L}(A^{0} - 2F_L)}{1 + K^{P}_{L} C^{0}_{L}} C^{0}_{L}. \quad (15)$$

Finally, by plugging this into equation *14* we can arrive at a quadratic equation for F_L,

$$F_L = \frac{K^{F}_{L} K^{P}_{L}(A^{0} - 2F_L)}{1 + K^{P}_{L} C^{0}_{L}} C^{0}_{L} \left(A^{0} - \frac{K^{P}_{L}(A^{0} - 2F)}{1 + K^{P}_{L} C^{0}_{L}} C^{0}_{L} - 2F_L\right). \quad (16)$$

This can be solved to find

$$F_L = \frac{4A^{0} K^{F}_{L} K^{P}_{L} C^{0}_{L} + (1 + K^{P}_{L} C^{0}_{L})^{2} - (1 + K^{P}_{L} C^{0}_{L})\sqrt{x_L}}{8 K^{F}_{L} K^{P}_{L} C^{0}_{L}}, \quad (17)$$

where x_L is defined to be

$$x_L \equiv 8A^{0} K^{F}_{L} K^{P}_{L} C^{0}_{L} + (1 + K^{P}_{L} C^{0}_{L})^{2}.$$

Equation *17* can be solved numerically for any given set of parameters, and doing this gives the expected non-monotonic relationship (supplementary Figure 2A) [47,48].

We note that the quadratic equation *16* has two solutions with a negative or positive sign for the square root in equation *17*. However, taking the positive solution results in values for F_L

that are higher than $A^0/2$ and thus this solution is not biologically relevant. We therefore focus on the negative solution for the square root.

The ALB model for ligand binding by a heterodimer receptor

A third model we consider in the paper is based on an architecture with two different types of receptor subunits that heterodimerize upon ligand binding.

We consider a sequential complex formation with a ligand, L, at a concentration C_L, that binds first to a specific receptor type, A, to form a partial complex, P_L. As a second step, the partial complex further binds a second receptor subunit, B, to form the full complex F_L. As with the previous models, we assume that the binding is reversible with first-order kinetics. The forward and reverse binding rates for the formation of the partial complexes, $k^P_{f\,L}$ and k^P_{rL}, and full complexes $k^F_{f\,L}$ and, $k^F_{r\,L}$, are considered as intrinsic properties of the specific ligand variant. Using these notations, the reactions can be summarized as (cf equations *7,8*):

$$A + L \underset{k^P_{rL}}{\overset{k^P_{fL}}{\rightleftharpoons}} P_L \quad (18)$$

$$B + P_L \underset{k^F_{rL}}{\overset{k^F_{fL}}{\rightleftharpoons}} F_L \quad (19)$$

We further denote, as before, the initial ligand concentration by C^0_L, which is considered to remain constant. A, B, P_L and F_L denote the number of receptors and complexes on the surface of a cell. We can thus write the dynamical equations describing these reactions as:

$$\frac{dA}{dt} = k^P_{rL} P_L - k^P_{fL} A C^0_L \quad (20)$$

$$\frac{dB}{dt} = k^F_{rL} F_L - k^F_{fL} P_L B \quad (21)$$

$$\frac{dP_L}{dt} = k^P_{fL} A C^0_L + k^F_{rL} F_L - k^P_{rL} P_L - k^F_{fL} P_L B \quad (22)$$

$$\frac{dF_L}{dt} = k^F_{fL} P_L B - k^F_{rL} F_L \quad (23)$$

As discussed previously, considering conservation of mass, and denoting the initial values of the receptors A^0 and B^0 we obtain

$$A^0 = A + P_L + F_L$$

$$A = A^0 - P_L - F_L, \quad (24)$$

$$B^0 = B + F_L$$

$$B = B^0 - F_L. \quad (25)$$

We consider our system in steady state, such that all time derivatives vanish, and the system can be reduced to two equations, which can be solved to give

$$P_L = K^P_L A C^0_L, \quad (26)$$

$$F_L = K^F_L P_L B \quad (27)$$

where K^P_L and K^F_L are defined as $K^P_L \equiv \frac{k^P_{fL}}{k^P_{rL}}$ and $K^F_L \equiv \frac{k^F_{fL}}{k^F_{rL}}$, describing the affinity of the ligand to subunit A, and the affinity of the partial complex to subunit B, respectively. Plugging equation *24* into equations *26*, we obtain the P_L dependence on the parameters in steady state:

$$P = K^P_L C^0_L (A^0 - P_L - F_L),$$

$$P_L = \frac{K^P_L (A^0 - F_L)}{1 + K^P_L C^0_L} C^0_L. \quad (28)$$

This can be plugged into equation *27* together with equation *25* and give rise to a quadratic equation for F_L:

$$F_L = \frac{K^F_L K^P_L (A^0 - F_L)(B^0 - F_L)}{1 + K^P_L C^0_L} C^0_L. \quad (29)$$

Solving equation *29* we get

$$F_L = \frac{K^F_L K^P_L C^0_L (A^0 + B^0) + 1 + K^P_L C^0_L - \sqrt{y_L}}{2 K^F_L K^P_L C^0_L}, \quad (30)$$

where y_L is defined to be:

$$y_L \equiv (K^F_L K^P_L C^0_L)^2 (A^0 - B^0)^2 + 2 K^F_L K^P_L C^0_L (A^0 + B^0)(1 + K^P_L C^0_L) + (1 + K^P_L C^0_L)^2.$$

Equation *30* can, like in the case of the previous model, be solved numerically for any given set of parameters.

As above, we chose a specific sign for the square root in equation 30 to provide the biologically relevant solution where F_L does not increase beyond the level of A^0 or B^0.

Calculating the intracellular response

The models enable us to determine the amount of full complexes that are generated given a specific ligand, with given parameters. However, in many signaling pathways the binding of a ligand to its receptor is only the first in a series of steps that culminate in the cell's response to the ligand, usually in the form of changes in gene expression [3,5,8]. We consider a simplified model for the downstream signal transduction where the full complex, F_L, elicits gene expression at a specific rate, e_L. This parameter can depend on the identity of the complex and in particular, in our model it is determined by the ligand. The *on* and *off* rates for a specific ligand as well as the physical proximity of the two receptor subunits resulting from the ligand dependent interaction could affect e_L independently of other ligand dependent parameters.

Given e_L, we can calculate a cell's response, E_L, as a function of the amount of full complexes formed assuming linear dependence:

$$E_L = e_L F_L \quad (31)$$

By plugging the solutions for equations *6*, *17* and *30* into equation *31*, we can solve for the ligand dependent cell response of the different receptor architecture models.

Local Scaling

Calculating local scaling

In order to quantify the effect of ligand parameters on the distribution of responses one needs to assume a specific distribution of receptors in the population and perform full simulations on the entire population. However, we aimed to define a local measure that would enable us to calculate a metric for a single cellular configuration that would provide a handle on the global diversity in the response. To achieve this, we defined a basic metric of local scaling (*S*) describing the local power law dependence of the response on the receptor amount:

$$E \sim A^S.$$

For infinitesimal changes, the local scaling can be calculated as the derivative of log(*E*) as a function of log(A):

$$S_L = \frac{\partial log(E)}{\partial log(A)}. \quad (32)$$

Intuitively, this metric measures the variability in ligand induced cell responses, *E* as we vary specific parameters, in particular the receptor levels on the cell membrane. We further wanted this metric to reflect effects of relative changes in receptor levels on relative changes in the response.

$$S = \frac{\frac{\Delta E}{E}}{\frac{\Delta A}{A}} = \frac{\Delta E}{\Delta A}\frac{A}{E} \rightarrow \frac{\partial E}{\partial A}\frac{A}{E} = \frac{\partial log(E)}{\partial log(A)}.$$

As the exponent in the power law increase, the same variability in the distribution of receptors will be translated into a higher dependence in the response of the system (Figure S1B). Thus, the local scaling metric can be used to estimate effects on the global variability at the population level.

Calculating scaling with respect to receptor amounts in the models

We first consider the AL model's response, given from plugging equation *6* into equation *31*

$$E_L = e_L \frac{K_L C^0_L}{1 + K_L C^0_L} A^0 \quad (33)$$

We can next break the calculation of the response's scaling with respect to *A* into two parts, first calculating the derivative of $\frac{\partial E_L}{\partial A^0}$, then its multiplication by $\frac{A^0}{E_L}$. Solving the derivative of $\frac{\partial E_L}{\partial A^0}$ we obtain

$$\frac{\partial E_L}{\partial A^0} = e_L \frac{K_L C^0_L}{1+K_L C^0_L} . \quad (34)$$

Next we multiply the derivative by $\frac{A^0}{E_L} = \frac{1+K_L C^0_L}{e_L K_L C^0_L A^0} A^0$, and obtain the scaling

$$S_L = e_L \frac{K_L C^0_L}{1+K_L C^0_L} \frac{1+K_L C^0_L}{e_L K_L C^0_L} = 1. \quad (35)$$

Thus, for the single unit receptor, the scaling is constant and independent of ligand parameters (see main text and Figure 2 for details). We note that since there is a simple linear relationship between E_L and A^0, a scaling of $S_L=1$ can be directly deduced. However, here we show the full derivation which can be directly extended to the other model.

In the same fashion we can calculate the scaling of the response of the ALA model with respect to changes in the initial subunit receptors amount. First, calculating the derivative of $\frac{\partial E_L}{\partial A^0}$ gives

$$\frac{\partial E_L}{\partial A^0} = e_L\left(\frac{1}{2} - \frac{1+K^P_L C^0_L}{2\sqrt{x_L}}\right). \quad (36)$$

Next we multiply by $\frac{A^0}{E_L} = \frac{8K^F_L K^P_L C^0_L A^0}{e_L\left(4A^0 K^F_L K^P_L C^0_L + (1+K^P_L C^0_L)^2 - (1+K^P_L C^0_L)\sqrt{x_L}\right)}$, as before, and obtain

$$S_L = \frac{4K^F_L K^P_L C^0_L A^0}{4A^0 K^F_L K^P_L C^0_L + (1+K^P_L C^0_L)^2 - (1+K^P_L C^0_L)\sqrt{x_L}} \left(1 - \frac{1+K^P_L C^0_L}{\sqrt{x_L}}\right) \quad (37)$$

Which can be numerically simulated for any given set of parameters. The x_L in equations *36* and *37* is defined in equation *17*.

Finally we calculated the scaling of the response in the ALB model with respect to changes in the total amount of the receptor subunits. In this case, however, as there are two different types of receptor subunits, we need to calculate the scaling with respect to each receptor separately. Starting with receptor subunit *A*, we can follow the same process as for the previous model, for which we obtain

$$S^A_L = \frac{K^F_L K^P_L C^0_L A^0}{K^F_L K^P_L C^0_L (A^0+B^0) + 1 + K^P_L C^0_L - \sqrt{y_L}} \left(1 - \frac{K^F_L K^P_L C^0_L (A^0-B^0) + 1 + K^P_L C^0_L}{\sqrt{y_L}}\right), \quad (38)$$

and following the same process for subunit *B*, we obtain

$$S^B_L = \frac{K^F_L K^P_L C^0_L B^0}{K^F_L K^P_L C^0_L (A^0+B^0) + 1 + K^P_L C^0_L - \sqrt{y_L}} \left(1 - \frac{K^F_L K^P_L C^0_L (B^0-A^0) + 1 + K^P_L C^0_L}{\sqrt{y_L}}\right). \quad (39)$$

Both can be numerically simulated for any given set of parameters. The y_L in both equations *38* and *39* is defined in equation *30*.

Importantly, we note that, based on the calculations, the scaling of the response to changes in the receptors or receptor subunits is independent of the activity rate, e_L, in all models.

Reducing parameter space using nondimensionalization

Our models include several parameters that determine the response of the system. For example, in the basic model for a single unit receptor we have four parameters, including three ligand parameters: concentration (C_L), affinity to the receptor (K_L) and activation rate (e_L), as well as a single cellular parameter, the amount of receptors (A^0). In order to fully determine the behavior of a model across all parameter values one needs to analyze the solution across the entire parameter space. Nondimensionalization is a standard method to reduce the number of parameters that should be independently varied in order to fully analyze the system [5]. When studying the biochemical parameters we should notice that they are dimensional, however the units can be chosen arbitrarily. This choice of units does not affect the normalized behavior of the system. For example changing the units of concentration from mg/ml to ng/ml would change the numeric value of C_L and K_L but will not change the resulting number of complexes.

More generally, in the AL model, C_L has units of ligand concentration and K_L has units of inverse ligand concentration. Changing these units by a scaling factor α will affect the values of concentrations and binding affinities in the following way:

$$C_L \rightarrow \alpha \cdot C_L \quad (40)$$

$$K_L \rightarrow \alpha^{-1} \cdot K_L.$$

However, such coordinated change will result in exactly the same values for the number of full complexes, F_L. While the value of each parameter is changed in the new unit scheme, the product $K_L C_L$ is dimensionless and will not change under redefinition of the units. From the equation for F_L (equation *6*) we see that the parameters only appear in this specific combination. Therefore, it is enough to only consider the case $K_L = 1$ (or alternatively $C_L = 1$) as other values can be mapped to this one by the transformation in equation *40*. Intuitively, we can always define the units of measurements for the ligand concentration such that the EC50 concentration is one. In these units K_L is equal to one, and the only relevant parameter is the ligand concentration. This process of nondimensionalization dictates that only these parameters will affect the behavior of the system. When scanning the parameter space we will thus only analyze such dimensionless parameter combinations. Overall for the AL model, there are three relevant parameters: $K_L C_L$, e_L and A^0.

Doing the same procedure for the ALA model, we start with four ligand parameters: C_L, $K^P{}_L$, $K^F{}_L$ and e_L, as well as one cellular parameter: A^0. In this case we consider the dimensionless combination $C_L K^P{}_L$, as before. In addition, by considering a similar change in the units of receptor amount, we get another dimensionless combination, $K^F{}_L A^0$. Overall the ALA model has three relevant parameters: $K^P{}_L C_L$, $K^F{}_L A^0$ and e_L.

Finally, for the ALB model, we start with four ligand parameters: C_L, $K^P{}_L$, $K^F{}_L$ and e_L, and two cellular parameters: A^0 and B^0. Using the same unit transformation for ligands and receptors, we will consider the following dimensionless parameters: $K^P{}_L C_L$, $K^F{}_L(A^0+B^0)$, A^0/B^0 and e_L.

Relationship between F_L and S_L in the ALA model

To determine the functional relationship between the full complex and the scaling in the ALA model we first define the following non dimensional parameters:

$$a \equiv A^0 K^F{}_L \quad (41)$$

$$c \equiv C^0{}_L K^P{}_L$$

$$f \equiv F_L K^F{}_L$$

$$X \equiv \sqrt{x_L}$$

Using these parameters,equation *17* can be rewritten as:

$$f = \frac{4ac+(1+c)^2-(1+c)X}{8c} \quad (42)$$

$$X^2 = 8ac + (1+c)^2.$$

Similarly, S_L (equation *37*) becomes:

$$S_L = \frac{4ac}{4ac+(1+c)^2-(1+c)X}\left(1 - \frac{1+c}{X}\right).$$

We consider the inverse of S_L:

$$\frac{1}{S_L} = \frac{4ac+(1+c)^2-(1+c)X}{4ac}\frac{X}{X-(1+c)}, \quad (43)$$

and, with some algebra, this can be rewritten as:

$$\frac{1}{S_L} = 1 + \frac{1+c}{8ac}\frac{2(1+c)X-8ac-2(1+c)^2}{X-(1+c)}. \quad (44)$$

Further development of equation *44* gives us:

$$\frac{1}{S_L} = 1 - \frac{(1+c)[X-(1+c)]}{8ac}$$

$$= \frac{8ac+(1+c)^2-(1+c)X}{8ac}$$

$$= \frac{4ac+(1+c)^2-(1+c)X}{8ac} + \frac{1}{2}. \quad (45)$$

Using equation 42, we find

$$\frac{1}{S_L} = \frac{f}{a} + \frac{1}{2}$$

$$= \frac{F_L}{A^0} + \frac{1}{2}. \quad (46)$$

Inverting this equation we find a simple dependence between S_L and F_L (Figure 3C):

$$S_L = \frac{2A^0}{2F_L+A^0} \quad .(47)$$

The scaling of the full complex across the parameter space of the ALA model

The metric defined in equation *32* is defined as local scaling of the number of full complexes, F_L, with the initial amount of receptor subunits, A^0. Here we will study the equation for F_L (equations *17* or *42*) to determine this scaling and its dependence on $K^F{}_L A^0$. We first consider the two regimes discussed in the main text, in both of which $c = 1$, such that the ligand is supplied at its EC50 (equation *44*). Under this condition we obtain:

$$f = \frac{a+1-\sqrt{2a+1}}{2}. \quad (48)$$

We study two regimes: $a = K^F{}_L A^0 \gg 1$ and $a = K^F{}_L A^0 << 1$, as described in the main text. For the first regime, $a \gg 1$, equation *48* can be simplified to be

$$f = \frac{a-\sqrt{2a}}{2}. \quad (49)$$

As $a \gg 1$, $a \gg \sqrt{2a}$. Thus, equation *52* can be further simplified to

$$f = \frac{a}{2},$$

$$F_L K^F{}_L = \frac{A^0 K^F{}_L}{2},$$

$$F_L = \frac{A^0}{2}. \quad (50)$$

Thus, when $K^F{}_L A^0 \gg 1$ the full complex F_L scales linearly with the initial amount of the receptor subunit A^0.

In the second regime, $a << 1$, we can expand equation *49* by using the taylor expansion for a square root. We find:

$$f = \frac{a+1-(1+a-\frac{a^2}{2})}{2},$$

$$= \frac{a^2}{4},$$

$$F_L K^F{}_L = \frac{(A^0 K^F{}_L)^2}{2},$$

$$F_L = \frac{K^F{}_L}{2} A^{0\,2}. \quad (51)$$

Thus, when $K^F{}_L A^0 << 1$, F_L scales quadratically with the number of receptors, A^0.

We can further extend our findings to a general ligand concentration. Here, under the first condition, when $K^F{}_L A^0 \gg (1 + K^P{}_L C^0{}_L)^2$, we obtain:

$$f = \frac{4ac-(1+c)\sqrt{8ac}}{8c}$$

$$= \frac{a}{2} - \frac{(1+c)}{\sqrt{8c}}\sqrt{a}. \quad (52)$$

In this regime, the equation can be approximated as

$$f = \frac{a}{2}, \quad (53)$$

so that

$$F_L K^F{}_L = \frac{A^0 K^F{}_L}{2},$$

$$F_L = \frac{A^0}{2}.\ (54)$$

Thus, we find that even in this more general case F_L scales linearly with A^0.

Next, when looking into the the second regime where $K^F{}_L A^0 << 1$, and expanding equation *42*, we obtain:

$$f = \frac{4ac+(1+c)^2-(1+c)\sqrt{(1+c)^2}\sqrt{1+\frac{8c}{(1+c)^2}a}}{8c},$$

$$= \frac{4ac+(1+c)^2-(1+c)^2[1+\frac{8c}{2(1+c)^2}a-\frac{1}{8}(\frac{8c}{(1+c)^2})^2a^2]}{8c}$$

$$= \frac{c}{(1+c)^2}a^2,$$

Which can be rewritten as

$$F_L = \frac{K^F{}_L K^P{}_L C^0{}_L}{(1+K^P{}_L C^0{}_L)^2}(A^0)^2.\ (55)$$

Thus, under the condition of $K^F{}_L A^0 << 1$, regardless of the $K^P{}_L C^0{}_L$, F_L scales quadratically with the number of receptors, A^0.

Unordered ligand binding in the ALB model

When modeling the ALB, heterodimeric receptor architecture, we assume that the assembly of the full complex is sequential. In this case, the ligand (*L*) first binds a specific receptor subunit type (*A*) and only then the partial complex binds the second receptor subunit type (*B*). While this is described as the mode of operation of pathways such as type I IFN, TGFβ, and BMP *in-vivo [4,5,39]*, under certain conditions the binding might proceed in parallel. Therefore, we consider the case of unordered binding in the ALB model. The resulting reactions can be summarized as:

$$A + L \underset{k_{rL}^{PA}}{\overset{k_{fL}^{PA}}{\rightleftharpoons}} P_L^A \quad (56)$$

$$A + L \underset{k_{rL}^{PB}}{\overset{k_{fL}^{PB}}{\rightleftharpoons}} P_L^B \quad (57)$$

$$A + P_L^B \underset{k_{rL}^{FA}}{\overset{k_{fL}^{FA}}{\rightleftharpoons}} F_L \quad (58)$$

$$B + P_L^A \underset{k_{rL}^{FB}}{\overset{k_{fL}^{FB}}{\rightleftharpoons}} F_L \quad (59)$$

with C_L being the ligand concentration as before, and $P^A{}_L$ and $P^B{}_L$ being the partial complexes generated through *L* binding either *A* or *B* respectively. The new binding reactions are considered to have first-order kinetics. Here, $k^{PA}{}_{f\,L}$ and $k^{PA}{}_{r\,L}$ are the forward and reverse binding rates of the ligand to receptor subunit *A* respectively, and similarly $k^{PB}{}_{f\,L}$ and $k^{PB}{}_{r\,L}$ are the forward and reverse binding rates of the ligand to receptor subunit *B*. Likewise, $k^{FA}{}_{f\,L}$ and $k^{FA}{}_{r\,L}$ are the forward and reverse binding rates, respectively, of P^B and subunit *A*, while $k^{FB}{}_{f\,L}$

and $k^{FB}{}_{rL}$ are the forward and reverse binding rates, respectively, of P^A and subunit B. These parameters are all intrinsic properties of the ligand.

As before, assuming steady state and using equation *2*, we find the following three equations:

$$0 = k^{PA}{}_{fL} A C^0{}_L + k^{FB}{}_{rL} F_L - k^{PA}{}_{rL} P^A{}_L - k^{FB}{}_{fL} P^A{}_L B, \quad (60)$$

$$0 = k^{PB}{}_{fL} B C^0{}_L + k^{FA}{}_{rL} F_L - k^{PB}{}_{rL} P^B{}_L - k^{FA}{}_{fL} P^B{}_L A, \quad (61)$$

$$0 = k^{FA}{}_{fL} P^A{}_L B - k^{FA}{}_{rL} F_L + k^{FB}{}_{fL} P^B{}_L A - k^{FB}{}_{rL} F_L. \quad (62)$$

As there is no energy invested in the formation of these complexes, e.g, in the form of ATP hydrolysis, this indicates that the kinetic rates satisfy detailed balance relation. Defining the affinities as

$$K^{PA}{}_L \equiv \frac{k^{PA}{}_{fL}}{k^{PA}{}_{rL}},\ K^{PB}{}_L \equiv \frac{k^{PB}{}_{fL}}{k^{PB}{}_{rL}},\ K^{FA}{}_L \equiv \frac{k^{FA}{}_{fL}}{k^{FA}{}_{rL}},\ K^{FB}{}_L \equiv \frac{k^{FB}{}_{fL}}{k^{FB}{}_{rL}},$$

detailed balance can be written as

$$K^{PA}{}_L \cdot K^{FB}{}_L = K^{PB}{}_L \cdot K^{FA}{}_L.$$

With this we obtain:

$$P^A{}_L = K^{PA}{}_L A C^0{}_L, \quad (63)$$

$$P^B{}_L = K^{PB}{}_L B C^0{}_L, \quad (64)$$

$$F_L = K^{FB}{}_L P^A{}_L B, \quad (65)$$

$$F_L = K^{FA}{}_L P^B{}_L A. \quad (66)$$

The condition of detailed balance also dictates that one of the above equations is redundant. As such, we can remove equation *65*.

Next, considering conservation of mass, and denoting the initial values of the receptors A^0 and B^0 we obtain

$$A^0 = A + P^A{}_L + F_L$$

$$A = A^0 - P^A{}_L - F_L, \quad (67)$$

$$B^0 = B + P^B{}_L + F_L$$

$$B = B^0 - P^B{}_L - F_L. \quad (68)$$

By plugging equations *67* and *68* into equations *63*, *64* and *66*, we can obtain the dependence of the two partial complexes' behavior on the parameters in steady state:

$$P^A{}_L = \frac{K^{PA}{}_L (A^0 - F_L)}{1 + K^{PA}{}_L C^0{}_L} C^0{}_L, \quad (69)$$

$$P^B{}_L = \frac{K^{PB}{}_L (B^0 - F_L)}{1 + K^{PB}{}_L C^0{}_L} C^0{}_L. \quad (70)$$

Finally, by plugging these into equation *66* we can arrive at a quadratic equation for F_L,

$$F_L = \frac{K^{FA}{}_L K^{PB}{}_L (B^0 - F_L)}{1 + K^{PB}{}_L C^0{}_L} C^0{}_L \left(A^0 - \frac{K^{PA}{}_L (A^0 - F_L)}{1 + K^{PA}{}_L C^0{}_L} C^0{}_L - F_L\right). \quad (71)$$

This can be solved to find

$$F_L = \frac{K^{FA}_L K^{PB}_L C^0_L (A^0 + B^0) + (1 + K^{PA}_L C^0_L)(1 + K^{PB}_L C^0_L) - \sqrt{z_L}}{2K^{FA}_L K^{PB}_L C^0_L}, \; (72)$$

where z_L is defined to be:

$$z_L \equiv (K^{FA}_L K^{PB}_L C^0_L)^2 (A^0 - B^0)^2 + 2K^{FA}_L K^{PB}_L C^0_L (A^0 + B^0)(1 + K^{PA}_L C^0_L)(1 + K^{PB}_L C^0_L) + [(1 + K^{PA}_L C^0_L)(1 + K^{PB}_L C^0_L)]^2 .$$

Equation *72* can, like in the case of the previous models, be solved numerically for any given set of parameters.

Next, using the same process described above, we can find the scaling for this model with respect to changes in the initial amount of the receptor subunits. First to subunit *A*:

$$S^A_L = \frac{K^{FA}_L K^{PB}_L C^0_L A^0}{K^{FA}_L K^{PB}_L C^0_L (A^0 + B^0) + (1 + K^{PA}_L C^0_L)(1 + K^{PB}_L C^0_L) - \sqrt{z_L}} \left(1 - \frac{K^{FA}_L K^{PB}_L C^0_L (A^0 - B^0) + (1 + K^{PA}_L C^0_L)(1 + K^{PB}_L C^0_L)}{\sqrt{z_L}}\right) \; (73)$$

And following the same process for subunit *B*, we obtain

$$S^B_L = \frac{K^{FA}_L K^{PB}_L C^0_L B^0}{K^{FA}_L K^{PB}_L C^0_L (A^0 + B^0) + (1 + K^{PA}_L C^0_L)(1 + K^{PB}_L C^0_L) - \sqrt{z_L}} \left(1 - \frac{K^{FA}_L K^{PB}_L C^0_L (B^0 - A^0) + (1 + K^{PA}_L C^0_L)(1 + K^{PB}_L C^0_L)}{\sqrt{z_L}}\right) \; (74)$$

Solving these equations for different parameters, we can show that the order in which the ligand binds the receptor subunit changes neither the range of the different scalings (supplementary Figure 3G), nor the molecular mechanism behind the diversity in scaling values.

Nonlinear dependence of the response on the number of full complexes

In all models we assume a linear dependence e_L between the response E_L and the full complex F_L, such that $E_L = e_L F_L$. This assumption has been held before, when modeling various ligand dependent responses of such pathways as Tgfβ and BMP [5,39]. However, cells' responses to a ligand aren't necessarily linear. Often, the interaction of downstream proteins or the cooperative binding of transcription factors results in a non-linear power-law dependence. It is thus interesting to consider how a nonlinear response would affect S_L.
Accordingly, we will consider a nonlinear relationship between E_L and F_L through an exponent *h.* As this is an intracellular property, it would generally not depend on the identity of the ligand. We thus have:

$$E_L = e_L (F_L)^h . \; (75)$$

In this case, based on equation *32*, S_L can be calculated as:

$$S_L = \frac{\partial E}{\partial A}\frac{A}{E} = \frac{\partial e_L (F_L)^h}{\partial A}\frac{A}{e_L}(F_L)^{-h} . \; (76)$$

Further expanding the derivative, we obtain

$$S_L = \frac{\partial F_L}{\partial A} e_L h (F_L)^{h-1} \frac{A}{e_L}(F_L)^{-h} . \; (77)$$

Which can be simplified to

$$S_L = h\frac{\partial(e_L F_L)}{\partial A} A(e_L F_L)^{-1} = h\frac{\partial \hat{E}}{\partial A}\frac{A}{\hat{E}} = h\hat{S}_L \text{. (78)}$$

Here:

$$\hat{E} = e_L F_L$$

$$\hat{S}_L = \frac{\partial \hat{E}}{\partial A}\frac{A}{\hat{E}}$$

are the expressions for the linear case. Thus, the nonlinear dependency of the response on the full complex results in a multiplicative factor for S_L. Considering the capacity to give rise to variation in the scaling, this factor does not affect the conclusion of the model. For the AL model this results in a larger scaling value, but still constant across all ligand parameters. For the ALA model this also results in a larger value, but still gives a two-fold range for the scaling. Finally for the ALB model, the maximal scaling would increase, but the minimal scaling value would remain 0.

Ligand induced degradation in the AL model

In all models tested, we assume a constant amount of receptors. However, many cells regulate the amount of their receptors through degradation. Furthermore, to regulate cell response, the binding of a ligand to a receptor may result in an induced internalization or degradation of that complex. In addition, this complex internalization and degradation rates could be ligand-specific. To test the effect of both constant and ligand-induced degradation of receptors on S_L we expanded the AL model. We can write the expanded dynamical equation as

$$\frac{dF}{dt} = k_{f\,L} A C^0_L - k_{r\,L} F_L - k_{lid\,L} F_L \text{ (79)}$$

Where the forward binding rate $k_{f\ L}$ and the reverse binding rate $k_{r\ L}$, as well as the ligand-induced degradation rate $k_{lid\ L}$, are intrinsic properties of the ligand, as above. Furthermore, we can write the dynamical equation for the receptor *A*

$$\frac{dA}{dt} = k_{r\,L} F_L - k_{f\,L} A C^0_L + \alpha - k_{cd\,L} A \text{ (80)}$$

where **α** is the production rate and k_{cd} is the degradation rate.
Assuming steady state as before, all time derivatives become zero, and equations *79* and *80* can be solved to give

$$F_L = \frac{k_{fL} C^0_L A}{k_{rL} + k_{lidL}} \text{ (81)}$$

$$A = \frac{\alpha + k_{rL} F_L}{k_{f\,L} C^0_L + k_{cd\,L}} \text{ . (82)}$$

In this model the number of receptors is changing. We define the receptor level as the amount of receptors in steady state without addition of ligands. This can be found from the steady state of eq. 80, setting the ligand concentration and number of complexes to zero. We get

$$0 = \alpha - k_{cd} A \text{ (83)}$$

Which can be written as

$$\alpha = k_{cd} A \rightarrow A^0 = \frac{\alpha}{k_{cd}} \text{ (84)}$$

where A^0 is the initial receptor level.

Plugging equations *82* and *84* into equation *81*, we obtain

$$F_L = \frac{k_{fL} C^0_L}{k_{rL} + k_{lidL}} \frac{k_{cd} A^0 + k_{rL} F_L}{k_{fL} C^0_L + k_{cdL}}$$

$$F_L = \frac{k_{fL} C^0_L k_{cd}}{(k_{rL} + k_{lidL})(k_{fL} C^0_L + k_{cd}) - k_{fL} C^0_L k_{rL}} A^0. \quad (85)$$

Thus, like in equation *6* and under the original assumption of no degradation, the complex F_L is linearly dependent on A^0, meaning that S_L is constant, as in the original model without internalization. Thus, in the case of the AL model, induced degradation or otherwise doesn't affect the response heterogeneity.

The LAAL model for ligand binding by a homodimer receptor

While our main focus when considering an architecture with two identical receptor subunits is the ALA model, we note that another architecture exists for ligand binding with homodimeric receptors, where two ligands bind together with two receptors. This architecture is used, for example in the FGF pathway. We denote this heterotetrameric model as the LAAL model. We consider a ligand *L*, at a concentration of C_L that binds to a receptor subunit, denoted by *A*, and forms a partial complex, P_L (Figure S2B). This partial complex further binds a second partial complex to form the full complex F_L. This is opposed to the ALA model where P_L binds another subunit *A*. As before, we assume that the binding is reversible with first-order kinetics. We consider the forward and reverse binding rates for the formation of the partial complexes, k^P_{fL} and k^P_{rL}, and full complexes k^F_{fL} and k^F_{rL}, to be intrinsic properties of the specific ligand variant. These reactions can be summarized as

$$A + L \underset{k^P_{rL}}{\overset{k^P_{fL}}{\rightleftharpoons}} P_L \quad (86)$$

$$P_L + P_L \underset{k^F_{rL}}{\overset{k^F_{fL}}{\rightleftharpoons}} F_L \quad (87)$$

(note that the eq. 86 is equivalent to eq. 7).

We simulated the model using the EQTK toolbox [49,50], an optimized Python-based numerical solver for biochemical reaction systems. Varying the ligand concentration, ligand receptor affinity as well as the receptor amount and partial complex affinity, we find that much like the ALA model, the full complex amount F_L varies from zero to half of the initial receptor amount (Figure S2C), and that S_L varies twofold, from one to two (Figure S2D). We thus conclude that the LAAL model show similar pattern of heterogeneity control as the LAL model.

The AL model for ligand binding by a single-unit receptor architecture with non-constant ligand concentration

In all models above we assume a constant ligand concentration (equation *2*), resulting from an assumption of a very large volume of media, such that the total amount of ligand molecules is much larger than that of the receptors. We emphasize that this does not require saturating levels of ligands, as even for small concentrations, the number of molecules diffusing in a large volume could be large. However, this assumption might not hold in all *in vivo* context, where the number of ligand molecules might diffuse in a limited space and their number could be lower or equal to the amount of receptors. As such, we opted to test how scaling will be affected by a non-constant ligand concentration, starting with the AL model of one monomeric receptor.

Under these conditions, and given the conservation of mass, the ligand amount C_L (now measured in molecules) becomes

$$C_L = C^0_L + F_L, \quad (88)$$

with C^0 denoting the initial ligand amount and F_L denoting the full complex, as before. Plugging equation *88* into equation *6* we obtain

$$F_L = K_L(C^0_L - F_L)(A^0 - F_L)$$

$$F_L = \frac{K_L(A^0+C^0_L)+1-\sqrt{z_L}}{K_L}, \quad (89)$$

where z_L is defined to be

$$z_L \equiv (K_L A^0 - K_L C^0_L)^2 + 1 + 2K_L(A^0 + C^0_L) .$$

We note that F_L is no longer linearly dependent on A^0, and as such we expect that S_L will no longer be a constant. Indeed, when calculating S_L, as described above, we obtain

$$S_L = \frac{K_L A^0}{K_L(A^0+C^0_L)+1-\sqrt{x_L}}\left(1 - \frac{1+K_L(A^0-C^0_L)}{\sqrt{z_L}}\right) \quad (90)$$

Which can be numerically simulated for any given set of parameters.

When simulating S_L under different initial receptor and ligand concentrations, as well as different ligand-receptor affinities, we noted that S_L remains constantly equal to one as long as the initial ligand amount is much larger than the initial receptor amount, regardless of the ligand's affinity to the receptor (Figure S5A). Furthermore, S_L starts to lower, and reaches zero when the initial ligand amount becomes equal or lower than the initial receptor amount and the affinity becomes much higher than the ligand amount (Figure S5A). Mechanistically, this can be explained as follows: As long as there is free ligand, the amount of full complex is linearly dependent on receptor *A*, like in the original AL model, with constant ligand concentration, and *S* will remain constant. This will always be the case as long as the initial ligand concentration is higher than the initial amount of receptors, or, in case it's equal or lower, as long as the affinity of the ligand to the receptor is lower than the ligand initial concentration. However, once there is no more free ligand, adding or removing receptor *A* won't change the amount of full complexes, making *S* equal zero. This will happen when there are more receptors than ligands, when the ligand's initial concentration C^0 is lower than

the initial amount of receptors A^0, and its affinity is sufficiently high. This makes the ligand, and not the receptor, the limiting factor for the model.

We note that this mechanism is very similar to the molecular mechanism behind the ALB model, where the range of sensitivity to receptor *A* depends on *A*'s ratio to receptor *B*, and vice versa (see supplementary figure 4 and main text for details). We thus hypothesized that in the ALB model the sensitivity for each receptor, *A* and *B*, will keep its range between zero and one, depending now on the ratio between the two receptors as well as the ratio to the initial ligand concentration C^0. For example, the sensitivity toward *A* will remain one as long as there are free receptor *B*s and free ligand, but will become zero once either of them is completely part of a complex. To test this we used EQTK [49,50] to solve the ALB model with limited ligand concentration, and initial concentration of the ligand, as well as initial receptor amounts set to one. As expected (Figure S5B) the ranges of S_A and S_B remain set between zero and one, depending on the aforementioned ratios, just like in the ALB model with constant ligand concentration.
